# Comprehensive profiling of RNA modification-related genes identifies RNA m^7^G binding protein CBP20 as a therapeutic target for tumor growth inhibition

**DOI:** 10.1038/s12276-025-01531-z

**Published:** 2025-09-01

**Authors:** Yoonsung Cho, Sang Eun Lee, Jaeik Oh, Dongjun Jang, Seungjae Shin, Soo-Jin Lee, Jiwon Kim, Yoojin Yang, Dohee Kim, Hae Rim Jung, Yumi Oh, Young Bin Park, Jae-Mun Choi, Sung-Yup Cho

**Affiliations:** 1 Department of Medicine, Seoul National University College of Medicine, Seoul, Republic of Korea; 2 Department of Biomedical Sciences, Seoul National University College of Medicine, Seoul, Republic of Korea; 3 Department of Translational Medicine, Seoul National University College of Medicine, Seoul, Republic of Korea; 4 Medical Research Center, Genomic Medicine Institute, Seoul National University College of Medicine, Seoul, Republic of Korea; 5 Calici Co., Ltd., San Jose, CA USA; 6 Calici Co., Ltd., Daejeon, Republic of Korea; 7 Department of Food and Biotechnology, Korea University, Sejong, Republic of Korea; 8 Department of Bio AI Convergence, Chungnam National University, Daejeon, Republic of Korea; 9 Cancer Research Institute, Seoul National University, Seoul, Republic of Korea

**Keywords:** Cancer genomics, Targeted therapies

## Abstract

RNA modifications add a crucial layer to gene expression regulation, though the roles of many RNA modification-related genes in cancer remain largely unexplored. Here we profile 76 RNA modification-associated genes across nine distinct types of modification (*N*^1^-methyladenosine, 5-methylcytosine, *N*^6^,2′-*O*-dimethyladenosine, 2′-*O*-dimethyladenosine, *N*^7^-methylguanosine, pseudouridine, uridylation, 2′-*O*-methylation, *N*^4^-acetylcytidine and adenosine-to-inosine editing) in four cancer types—breast, colon, liver and lung—through a comprehensive analysis of The Cancer Genome Atlas data. Our analysis identified three candidate genes with increased expression in cancer tissues, with elevated levels associated with poor survival across multiple cancer types: the 5-methylcytosine methyltransferases NSUN2 and DNMT3B and CBP20, an *N*^7^-methylguanosine binding protein. Of these, CBP20 emerged as a key candidate, with its knockdown leading to reduced cancer cell viability, apoptosis induction and G1–S cell cycle arrest. RNA sequencing further confirmed the downregulation of cell-cycle-related pathways upon CBP20 depletion. Through a signature similarity search using the Library of Integrated Network-Based Cellular Signatures dataset, we identified raloxifene, purpurogallin and enoxacin as pharmacological agents that mimic the effects of CBP20 knockdown. Treatment with these agents significantly inhibited cell growth, highlighting a potential avenue for targeted cancer therapy. These findings suggest that CBP20 plays a pivotal role in RNA modification-mediated tumor progression and may represent a promising therapeutic target in cancer treatment.

## Targeting CBP20 offers new hope for cancer therapy

Cancer cells often have unique patterns of gene expression due to changes in RNA. In this study, researchers focused on RNA modifications, which are small chemical changes to RNA that can affect how it works. They found that certain genes related to RNA modifications were more active in cancer cells than in normal cells. They identified three key genes—NSUN2, DNMT3B and CBP20—that were linked to poor survival in patients with cancer. These genes are involved in cell cycle regulation, which controls how cells grow and divide. By reducing the activity of these genes in lab-grown cancer cells, they observed slower cell growth and increased cell death. The study concludes that targeting RNA modifications could be a new way to treat cancer. Future research may explore RNA modification-targeting drugs to understand their potential as cancer therapies.

This summary was initially drafted using artificial intelligence, then revised and fact-checked by the author.

## Introduction

The characteristics of a cell are determined by the intricate and specific patterns of gene expression, which collectively define its functional properties and overall phenotype. Cancer cells, which originate from specific mutational and genomic alterations, are also accompanied by the reprogramming of gene regulatory controls, resulting in distinct patterns of gene expression^1^. The unique patterns of gene expression have been employed for the diagnosis and classification of cancers, as well as for the identification of specific therapeutic targets. Furthermore, the dynamic regulation of gene expression is crucial for the survival of cancer cells in response to various external stress conditions, such as hypoxia, nutrient deprivation and chemotherapeutic drugs^2^. Gene expression is controlled by multiple layers of mechanisms, including epigenetic, transcriptional, post-transcriptional, translational and post-translational levels^2^. Within the array of regulatory mechanisms, the post-transcriptional modulation of mRNA metabolism, involving activities such as splicing, nuclear transportation and degradation, is a crucial regulatory nexus influencing cancer gene expression^3,4^. However, the precise molecular mechanisms governing this process remain relatively unexplored.

RNA metabolism is substantially influenced by post-transcriptional chemical modifications, such as *N*^6^-methyladenosine (m^6^A), 5-methylcytosine (m^5^C), *N*^1^-methyladenosine (m^1^A) and various others^5,6^. Distinct chemical modifications of RNA are controlled by proteins known as writers, responsible for depositing specific modifications, erasers, which remove these modifications, and readers, which recognize and bind the chemical alterations, thereby influencing RNA metabolism^5^. Chemical modifications of RNA nucleotides induce changes in the electrostatic charge, hydrophobic surface and base pairing properties of RNA molecules. These alterations affect the binding affinity of reader proteins to RNA, thereby modulating RNA metabolism, particularly impacting gene expression-related processes such as splicing, translation, stability and degradation^5^. These modifications not only govern mRNA metabolism but also influence other RNA types, including ribosomal RNA (rRNA), micro RNA (miRNA) and long non-coding RNA (lncRNA)^7^. Recent studies have revealed that alterations in RNA modifications within cancer cells and the tumor microenvironment result in the extended persistence of oncogenic RNA molecules, which should typically undergo degradation, while accelerating the degradation of tumor-suppressive RNAs^8–12^. Consequently, these modifications exert an influence on tumorigenesis, along with cancer-related phenomena such as invasion, migration, apoptosis, angiogenesis and cell proliferation^13,14^. Therefore, elucidating the intricate molecular mechanisms involved in this process is not only important for advancing our knowledge of cancer biology but also for developing novel strategies for cancer treatment.

Although numerous studies have investigated the significance and detailed mechanisms of m^6^A, the most prevalent RNA modification^15^, especially in the context of cancers, research on the roles of other RNA modifications in cancer remains limited. In this study, we explore the alterations in RNA modification-associated genes, excluding m^6^A, during carcinogenesis. To investigate how diverse RNA modifications differentially influence cancer development and to identify common genes influencing tumorigenesis across cancer types, we conducted a comparative analysis of gene expression associated with each RNA modification and their corresponding survival outcomes utilizing the The Cancer Genome Atlas (TCGA) database. Our study elucidated several key genes associated with RNA modification in cancer development, presenting a new avenue for cancer therapeutics through the manipulation of RNA modification processes.

## Materials and methods

### Selection of RNA modification-related genes

We analyzed the mRNA expressions of genes associated with a total of nine different types of RNA modification, including *N*^1^-methyladenosine (m^1^A), 5-methylcytosine (m^5^C), *N*^*6*^,2′-*O*-dimethyladenosine (m^6^Am), *N*^7^-methylguanosine (m^7^G), pseudouridine, uridylation, 2′-*O*-methylation (2′-*O*-Me), *N*^4^-acetylcytidine (ac^4^C) and adenosine-to-inosine editing (AI editing). The writer, eraser and reader genes for each RNA modification were selected based on previous reports^5,16–22^ (Supplementary Table 1).

### Differential gene expression between tumor and normal tissues

All mRNA expression data of genes associated with RNA modification were extracted from TCGA cohort (https://www.cancer.gov/ccg/research/genome-sequencing/tcga). The fold change of genes between normal and cancer tissues was calculated by the following formula:

$${{FC}}=\frac{{\rm{Mean}}\,{{\rm{expression}}}_{{\rm{tumor}}\; {\mathrm{tissue}}}}{{\rm{Mean}}\,{{\rm{expression}}}_{{\rm{nomal}}\; {\mathrm{tissue}}}}.$$

We used R software ‘pheatmap’ package to represent a heat map from the fold change data (https://cran.r-project.org/web/packages/pheatmap/index.html). In the heat map, red indicates genes that are more highly expressed in tumor tissues, while blue represents genes that are more highly expressed in normal tissues. The statistical difference was calculated by a *t*-test, and the statistical significance were denoted by asterisks (*P* ≥ 0.05: not significant, *P < 0.05, **P < 0.01, ***P < 0.001, ****P < 0.0001).

### Survival analysis

R software package ‘maxstat’ was employed to analyze survival data, optimizing the median survival difference between groups with high and low gene expression levels. When prognosis was poorer in the high-expression group compared with the low-expression group, with a *P* value less than 0.05, it was labeled as ‘worse in high’ and shown in red. Conversely, when prognosis was poorer in the low-expression group compared with the high-expression group, with a *P* value less than 0.05, it was labeled as ‘worse in low’ and shown in blue. Cases that did not fit into either category were labeled as ‘ns’ (not significant), with *P* values represented by asterisks, similar to the notation used for fold change.

### GSEA

A gene set enrichment analysis (GSEA) was conducted to elucidate the biological pathways associated with the altered gene expression. The software and data used for this analysis were obtained from the GSEA website (https://www.gsea-msigdb.org/gsea/index.jsp). We employed the R software package ‘fgsea’ and used hallmark gene sets to investigate the enriched gene sets in groups with high or low expression, as determined by maxstat.

### Cell culture

The human breast cancer cell line (MDA-MB-468), human liver cancer cell line (Huh7), human lung cancer cell line (A549), human normal colonocyte (CCD-18Co) and human normal lung fibroblast (MRC-5 and WI-38) were acquired from the Korean Cell Line Bank, while the human colorectal cancer cell line (HCT116) was purchased from Horizon Discovery. All cancer cell lines were cultured in RPMI-1640 medium (HyClone #SH30027), CCD-18Co cells were cultured in DMEM medium (HyClone #SH30243) and MRC-5 and WI-38 cells were cultured in MEM medium (HyClone #30024). All media were supplemented with 10% fetal bovine serum (FBS) (HyClone #SH30919) and 1% penicillin–streptomycin (Gibco #15140-122). The cells were subcultured with Dulbecco's phosphate buffered saline (DPBS) (Welgene #LB001-02) and 0.25% Trypsin-EDTA (Gibco #25200-056) every 2–3 days and maintained in a humidified cell incubator at 37 °C with 5% CO_2_. All cells used in the experiment were confirmed to be mycoplasma-free using an e-Myco Mycoplasma PCR Detection Kit (LiliF #25235).

### siRNA transfection

Control short interfering RNA (siRNA) (siNC, negative control DsiRNA, Integrated DNA Technologies (IDT) #51-01-14-04), siRNA for CBP20 (siCBP20, IDT hs.Ri.NCBP2.13.1, sense: 5′-rGrGrUrGrArUrUrUrCrUrCrUrCrUrArArUrArUrArUrCrUGA-3′, anti-sense: 5′-rUrCrArGrArUrArUrArUrUrArGrArGrArGrArArArUrCrArCrCrUrC-3′), siRNA for NSUN2 (siNSUN2, IDT hs.Ri.NSUN2.13.1, sense: 5′-rArArArGrUrUrGrUrArCrUrUrCrUrArUrCrArArGrArArACA-3′, anti-sense: 5′-rUrGrUrUrUrCrUrUrGrArUrArGrArArGrUrArCrArArCrUrUrUrUrG-3′), siRNA for DNMT3B (siDNMT3B, IDT hs.Ri.DNMT3B.13.1, sense: 5′-rArCrArArUrGrGrCrUrArArGrArUrArCrCrArArArArCrCAC-3′, anti-sense: 5′-rGrUrGrGrUrUrUrUrGrGrUrArUrCrUrUrArGrCrCrArUrUrGrUrUrU-3′), siRNA for METTL1 (siMETTL1, IDT hs.Ri.METTL1.13.1, sense: 5′-rGrCrArCrArArGrUrGrGrCrGrArArUrCrArUrCrArGrUrCCC-3′, anti-sense: 5′-rGrGrGrArCrUrGrArUrGrArUrUrCrGrCrCrArCrUrUrGrUrGrCrUrU-3′) and siRNA for RNMT (siRNMT, IDT hs.Ri.RNMT.13.1, sense: 5′-rArGrArArCrArGrUrCrArArGrUrArArGrGrUrUrArCrCrUTT-3′, anti-sense: 5′-rArArArGrGrUrArArCrCrUrUrArCrUrUrGrArCrUrGrUrUrCrUrUrC-3′) were synthesized by IDT. Transfection with siRNAs was performed using Lipofectamine 2000 Reagent (Invitrogen #11668019) following the standard instructions of the manufacturer. All cells for experiments except cell proliferation assay were seeded in six-well plates, and 60 pmol of siRNAs were used for transfection in each well. For the apoptosis assay, siRNAs were transfected for 48 h without changing media, while for the other experiments, media change was carried out or cells were reseeded with appropriate numbers after 24 h.

### Cloning of the CBP20 overexpression plasmid

Human CBP20 wild-type and mutant (Y20A, Y43A) cDNA gBlock were purchased from IDT and cloned into pCMV-3Tag-1A vector. All plasmids were verified by Sanger sequencing.

### Cell proliferation assay

A total of 2,000 cells were seeded in each well of a 96-well plate with 100 μl of media. Transfection was then performed at the same time without changing media, using 100 μl of Opti-MEM, 0.25 μl of Lipofectamine 2000 and 5 pmol of siRNA for each well. After transfection for the indicated time points, cell proliferation was measured by water-soluble tetrazolium salt (WST) using EZ-Cytox (DoGenBio #EZ-3000) following the manufacturer’s instructions. Each experiment was conducted in triplicate. After measuring the absorbance at 450 nm, the cells were collected with RLT lysis buffer for knockdown validation by quantitative real-time PCR.

### qRT–PCR

The cells were collected with RLT lysis buffer in the RNeasy Mini Kit (Qiagen #74106) and stored at −80 °C. RNA was isolated using the RNeasy Mini Kit according to the manufacturer’s instructions and reverse-transcribed into cDNA using PrimeScript RT Master Mix (Perfect Real Time) (Takara #RR036A) with the standard protocol. Real-time PCR was performed with a CFX96 Real-Time System (Bio-Rad) and the reaction solution in each well was composed of 10 μl iQ SYBR Green Supermix (Bio-Rad #1708882), 2 μl forward primers (4 μM), 2 μl reverse primers (4 μM), 4 μl autoclaved triple distilled water and 2 μl cDNA. Each reaction was run in triplicate, and the primer sequences used for quantitative real-time PCR (qRT–PCR) were as follows: CBP20-F: 5′-GCCAGGTTCGGGATGAGTAT-3′, CBP20-R: 5′-GCTCGTGTGCAGACTTTAGG-3′, NSUN2-F: 5′-CAGTGGAGACGGCACTATGA-3′, NSUN2-R: 5′-GACACATCAGCAAGCTCCAA-3′, DNMT3B-F: 5′-TTGGAATAGGGGACCTCGTGTG-3′, DNMT3B-R: 5′-AGAGACCTCGGAGAACTTGCCA-3′, METTL1-F: 5′-AAACCCCGGACTGGGATCAT-3′, METTL1-R: 5′-CTGGCTTCACAGGGTAGCG-3′, RNMT-F: 5′-GTACTGATATTGCCGATGT-3′, RNMT-R: 5′-ATTCACTATCACGACGATT-3′, CDK2-F: 5′-GCTTGTTATCGCAAATGCTG-3′, CDK2-R: 5′-GGAGAGGGTGAGATTAGGGC-3′, CDK6-F: 5′-CTCCGAGGTCTGGACTTTCT-3′, CDK6-R: 5′-TGCTCTGTACCACAGCGTGA-3′, CCNA2-F: 5′-GGATGGTAGTTTTGAGTCACCAC-3′, CCNA2-R: 5′-CACGAGGATAGCTCTCATACTGT-3′, CCNB1-F: 5′-GCCTGAGCCTATTTTGGTTGATAC-3′, CCNB1-R: 5′-TCCATCTTCTGCATCCACATCA-3′, CCND3-F: 5′-CTGGCCATGAACTACCTGGA-3′, CCND3-R: 5′-GCCAGGAAATCATGTGCAAT-3′, CCNE2-F: 5′-ACTGATGGTGCTTGCAGTGA-3′, CCNE2-R: 5′-AAAATGGCACAAGGCAGCAG-3′, CDKN1A-F: 5′-GACCAGCATGACAGATTTC-3′, CDKN1A-R: 5′-TGAGACTAAGGCAGAAGATG-3′, CDKN1B-F: 5′-AGGACACGCATTTGGTGGA-3′, CDKN1B-R: 5′-TAGAAGAATCGTCGGTTGCAGGT-3′, GAPDH-F: 5′-GAAGGTGAAGGTCGGAGT-3′ and GAPDH-R: 5′-GAAGATGGTGATGGGATTTC-3′. The relative mRNA expression of each gene was normalized to that of GAPDH by calculating using the 2^−ΔΔCT^ method^23^.

### Colony formation assay

For knockdown experiments, a total of 1000 cells for HCT116, Huh7 and A549, and 1 × 10^4^ cells for MDA-MB-468 were reseeded in 60 mm culture dishes 24 h after siRNA transfection and cultured for 14 days with media changes every 2–3 days. For drug experiments, total 1000 cells were seeded in a 60 mm culture dish under various drug conditions and cultured for 14 days with fresh drug treatment every 3 days. After 2 weeks, the cells were washed with DPBS and fixed with methanol (Merck #1.06009.2511) for at least 30 min at room temperature. Colonies were stained with crystal violet solution (Merck #V5265) for 5 min at room temperature, then washed with triple distilled water. The cells were seeded in three dishes for every experiment, and the relative colony formation was analyzed by ImageJ.

### BrdU incorporation assay

A total of 1 × 10^5^ cells of MDA-MB-468, HCT116 and A549, and 8 × 10^4^ cells of Huh7 were seeded in a 12-well plate after transfection for 24 h with siRNA or 72 h with siRNA and plasmid. After the cells were attached to the plate, 10 μg ml^−1^ bromodeoxyuridine (BrdU) (Biolegend #423401) was added and treated for the indicated times. The cells were detached by 0.25% Trypsin-EDTA and DPBS with 2% FBS, then fixed with 70% ethanol. The cells were permeabilized in 2 N HCl with 0.5% Triton X-100 for 30 min and neutralized in 0.1 M sodium tetraborate for 10 min at room temperature. All samples, except for the unstained sample, were labeled with 0.4 μl Alexa Fluor 647 anti-BrdU (Biolegend #364108) in 100 μl DPBS with 1% FBS and 0.5% Tween-20 for 30 min at room temperature, while being protected from exposure to light. BrdU incorporation was measured by flow cytometry.

### Cell cycle analysis

After transfection for 24 h, the cells were reseeded in a six-well plate and trypsinized after 24 h. The cells were washed with cold DPBS twice and fixed with 70% ethanol. After fixation, cells were washed with DPBS twice and incubated with 5 μg ml^−1^ of 4′,6-diamidino-2-phenylindole (DAPI) (Sigma #D9542) in 100 μl of DPBS containing 0.5% Triton X-100 for 30 min at room temperature in the dark. The cell cycle was analyzed using flow cytometry.

### Apoptosis assay

After transfection for 48 h without a media change, all cells were collected and washed twice with cold DPBS. After diluting the 10× Annexin V Binding Buffer (BD Biosciences #556454) with autoclaved triple distilled water, the cells were stained with 100 μl staining volume including 5 μl FITC Annexin V (BD Biosciences #556419) and 5 μl 7-AAD viability staining solution (Biolegend #420404) with 1× Annexin V Binding Buffer for 15 min in the dark at room temperature. Appropriate volumes of 1× Annexin V Binding Buffer were added, and the cell death was analyzed with flow cytometry.

### Flow cytometry analysis

BrdU incorporation, cell cycle and apoptosis assay were analyzed by flow cytometry. Flow cytometry was performed using the BD LSRII (SORP) with applicable lasers, colors and FACS DIVA software (BD Biosciences). FlowJo 10.8.1 was used to analyze all the results obtained from the flow cytometer.

### Western blot

The cell pellet samples were lysed using RIPA buffer (ThermoFisher Scientific #89900) with a protease inhibitor cocktail (Roche #11 873 580 001) and phosphatase inhibitors (Roche #04 906 837 001) and incubated for 30 min on ice. After centrifuging the samples, supernatants were used for protein quantification using BCA protein assay kits (ThermoFisher Scientific #23225). All protein samples were boiled with 5× sample buffer (containing 60 mM 1 M Tris–HCl (pH 6.8), 25% glycerol, 2% SDS, 14.4 mM 2-mercaptoethanol and 0.1% bromophenol blue) for 10 min at 95 °C. The same amounts of proteins were loaded onto an SDS–polyacrylamide gel electrophoresis, and the separated proteins were transferred onto a nitrocellulose membrane (Cytiva #10600001, #10600002). The membranes were blocked with 5% skim milk (BD Difco #232100) dissolved in TBS-T (Tris buffered saline with Tween-20, Biosesang #TR2007-000-74) for more than 1 h at room temperature. Primary antibodies were diluted following the datasheets using a 5× antibody dilution solution (1% BSA, 0.1% sodium azide) diluted with TBS-T. After blocking, the membranes were incubated with the primary antibodies at 4 °C overnight and with horseradish peroxidase-conjugated secondary antibodies (ThermoFisher Scientific #31430, #31460) for 1 h at room temperature. The images were visualized using enhanced chemiluminescence (ECL) substrate (ThermoFisher Scientific #34580) and the Amersham Imager 600 (GE Healthcare). The antibodies used for the western blot are as follows: anti-CBP20 (SantaCruz #sc-137123), β-actin (Sigma #A5441), anti-CDK2 (Cell Signaling Technology #2546), anti-CDK4 (Cell Signaling Technology #12790), anti-CDK6 (Cell Signaling Technology #3136), anti-Cyclin A2 (Cell Signaling Technology #4656), anti-Cyclin B1 (Cell Signaling Technology #12231), anti-Cyclin D1 (Cell Signaling Technology #2978), anti-Cyclin D3 (Cell Signaling Technology #2936), anti-Cyclin E2 (Cell Signaling Technology #4132), anti-p21 (Cell Signaling Technology #2947) and anti-p27 (Cell Signaling Technology #3686).

### Drug cytotoxicity assay

A total of 5,000 cells were seeded in each well of a 96-well plate with increasing concentrations of raloxifene (MedChemExpress #HY-13738), purpurogallin (MedChemExpress #HY-12136) or enoxacin (Selleckchem #S1756). The cell viability was determined by WST after incubating with drugs for 72 h, following the instructions provided in the manual. Each experiment was carried out three times.

### RNA sequencing and analysis

MDA-MB-468, HCT116, Huh7 and A549 cells were transfected with control siRNA or siRNA targeting CBP20 for 24 h, followed by media replacement, and collected after a total of 48 h. Control and CBP20-knockdown samples were prepared in triplicate for subsequent analysis. Total RNA was extracted using the RNeasy Plus Mini Kit (Qiagen). RNA sequencing libraries were prepared from total RNA using the Illumina TruSeq mRNA prep kit. Sequencing was performed on an Illumina NovaSeq platform. FASTQ files were processed using Nextflow (v. 23.10.0). Differential gene expression analysis was conducted using DESeq2 (Bioconductor, DESeq2). GSEA was performed using differentially expressed genes (DEGs) with the fgsea (Bioconductor, fgsea), while gene expression heat maps were generated by calculating *Z*-scores from transcripts per million (TPM) values and visualized using pheatmap (pheatmap function, RDocumentation). Also, DEGs were utilized as input gene lists in SigCom LINCS (https://maayanlab.cloud/sigcom-lincs/#/SignatureSearch/UpDown)^24^ to search for substances with effects similar to CBP20 depletion. Chemical lists from LINCS L1000 Chemical Perturbations (2021) were obtained by uploading the gene lists and submitting to SigCom LINCS.

### RIP-sequencing and analysis

A549 cells were collected by washing twice with ice-cold DPBS and cell pellets were resuspended with 230 μl of lysis buffer (150 mM KCl, 50 mM Tris–HCl (pH 7.5), 2 mM EDTA, 0.5% NP-40, 0.5 mM dithiothreitol, protease inhibitor cocktail, 100 U ml^−1^ RNase inhibitor). The lysate was incubated on ice for 5 min and centrifuged for 10 min to get supernatant. Pierce Protein A/G Magnetic Beads (ThermoFisher Scientific #88803) for immunoprecipitation were prepared by washing with wash buffer (200 mM NaCl, 50 mM Tris–HCl (pH 7.5), 2 mM EDTA, 0.05% NP-40) and incubated with 10 μg antibody for more than 30 min at room temperature. A total of 10 μl of cell lysate was used as input, and 100 μl of cell lysate was incubated with beads of anti-CBP20 (SantaCruz) or IgG (Cell Signaling Technology #61656) at 4 °C overnight with 900 μl of immunoprecipitation buffer (200 mM NaCl, 50 mM Tris–HCl (pH 7.5), 2 mM EDTA, 0.05% NP-40, 0.5 mM dithiothreitol and 100 U ml^−1^ RNase inhibitor). The beads were then washed six times with wash buffer and eluted with 150 μl of elution buffer (117 μl of wash buffer, 15 μl of 10% SDS and 18 μl of 600 U ml^−1^ proteinase K per sample) at 55 °C for 30 min using thermomixer. RNA was then extracted using TRIzol LS Reagent (Invitrogen #10296010) according to the manufacturer’s instruction. Input and immunoprecipitation samples were used to generate the library using SMARTer Stranded RNA library preparation kit and sequenced using Illumina NovaSeq platform. FASTQ files were processed using Trimmomatic (version 0.39) for trimming adapter sequences. The trimmed data were aligned to the human reference genome (GRCh38_v46) using STAR (v2.7.11b). For peak calling, exomePeak2 R package (v1.16.2) was used and peaks were annotated using biomaRt R package (v2.62.1). GSEA homepage (https://www.gsea-msigdb.org/gsea/msigdb/human/annotate.jsp) was used to investigate human gene sets.

### In silico docking analysis of CBP20 and CBP80 with raloxifene, purpurogallin and enoxacin

In silico molecular docking analysis was conducted using the Pharmaco-Net platform (https://pharmaco-net.org)^25^ powered by Calici (https://calici.co) to investigate the binding interactions between CBP20 (PDB ID: 1H6K (Chain X)) and CBP80 (PDB ID: 1H6K (chain A)) with raloxifene, purpurogallin and enoxacin. The protein structures were uploaded in PDB format, and the ligand structures were retrieved from the PubChem database. Active site prediction was performed using the PocketFinder module, which automatically identified and refined the docking regions, correcting structural errors (for example, missing residues and alternative conformations). Docking simulations were executed using AI-Dock and DeepCalici-plus modules, generating binding energy values (in kilocalories per mole) and binding affinity estimates (in micromole) for each protein–ligand complex. Afterward, the InteractionViewer module was used to conduct a three-dimensional analysis to confirm the binding image between the protein(s) and the compound(s).

## Results

### Alterations in gene expression of RNA modification writers, erasers, and readers across cancer types

**Fig. 1:**
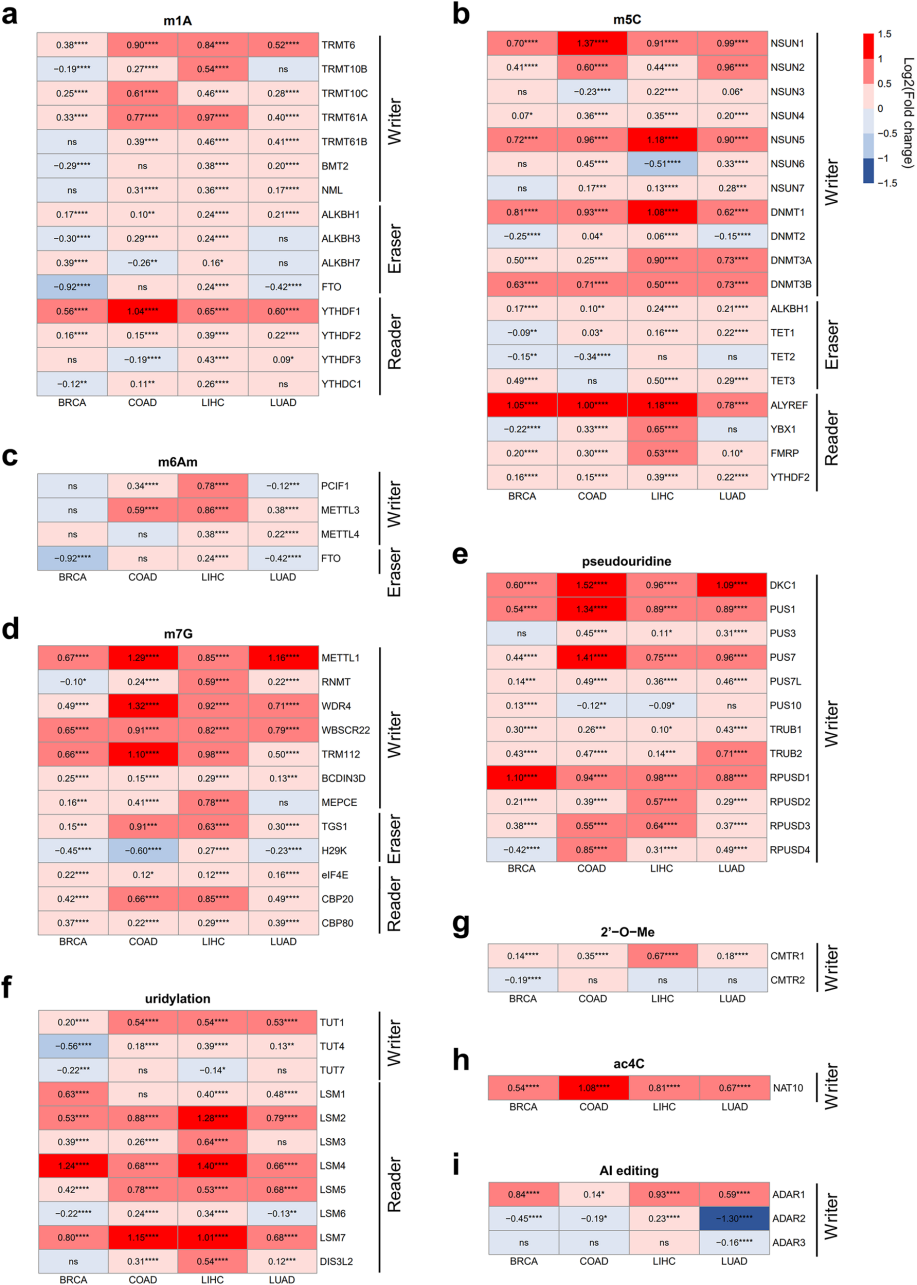
Alterations in gene expression of RNA modification writers, erasers and readers across cancer types. **a**–**i**, A heat map for gene expression of RNA modification-related genes across four cancer types; the RNA modification-related genes are categorized as follows: m^1^A (**a**), m^5^C (**b**), m^6^Am (**c**), m^7^G (**d**), pseudouridine (**e**), uridylation (**f**), 2′-*O*-Me (**g**), ac^4^C (**h**) and adenosine-to-inosine editing (AI editing) (**i**). Each column represents a cancer type, and each row corresponds to RNA modification-related genes. The box color indicates the log_2_(fold change) in gene expression between cancer and normal tissues from the TCGA database. The number in each box shows log_2_(fold change), with asterisks indicating the adjusted *P* value from a *t*-test (*P* ≥ 0.05: ns, *P < 0.05, **P < 0.01, ***P < 0.001, ****P < 0.0001). BRCA, breast invasive carcinoma; COAD, colon adenocarcinoma; LIHC, liver hepatocellular carcinoma; LUAD, lung adenocarcinoma.

**Fig. 2:**
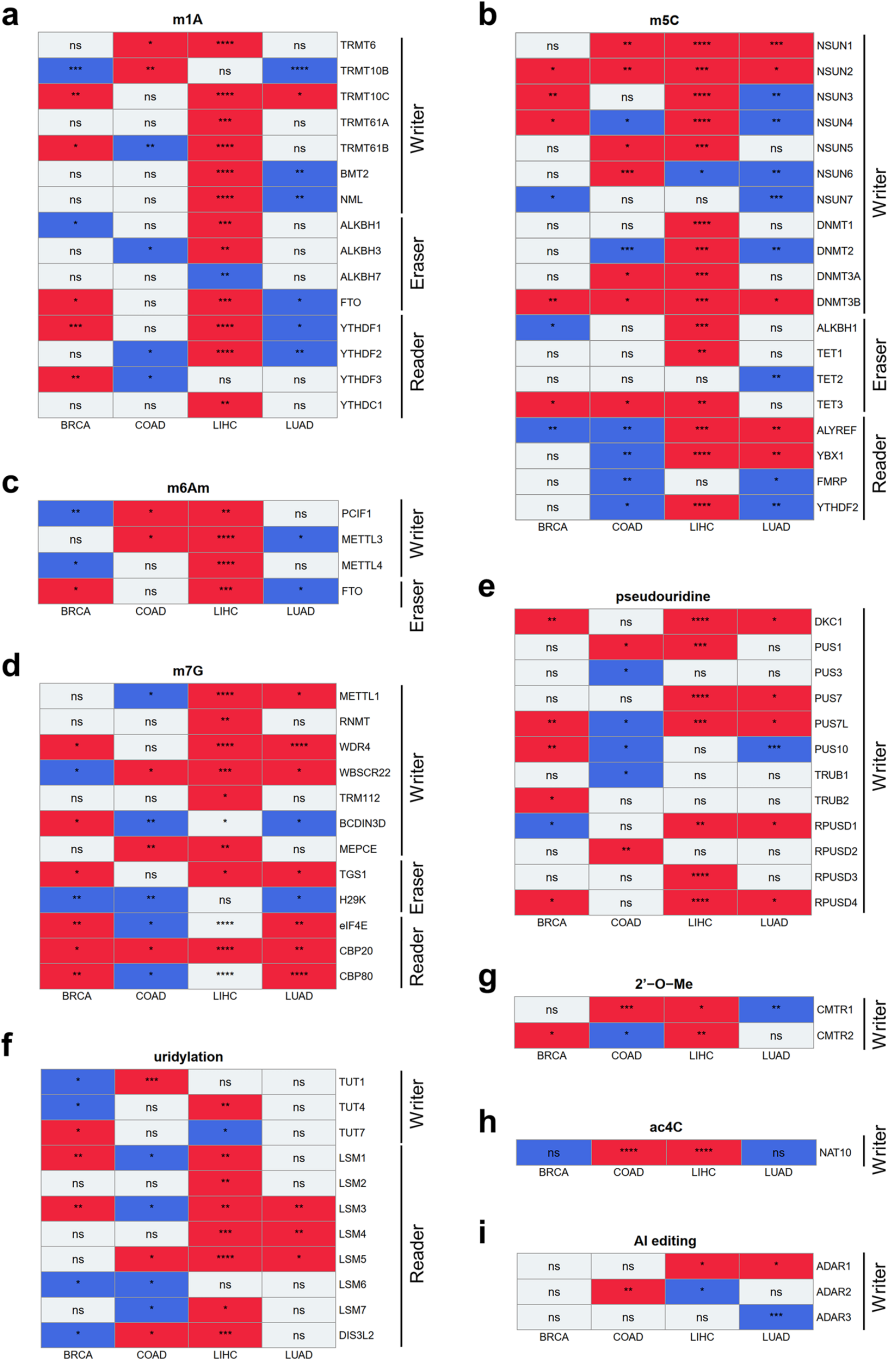
Prognostic effect of gene expression of RNA modification writers, erasers and readers across cancer types. **a**–**i** A heat map showing the prognostic impact of RNA modification-related genes across four cancer types; the RNA modification-related genes are categorized as follows: m^1^A (**a**), m^5^C (**b**), m^6^Am (**c**), m^7^G (**d**), pseudouridine (**e**), uridylation (**f**), 2′-*O*-Me (**g**), ac^4^C (**h**) and adenosine-to-inosine editing (AI editing) (**i**). Each column represents a cancer type and each row corresponds to RNA modification-related genes. The box color represents the prognostic effect of each gene. Red indicates poorer survival in patients with high gene expression compared with those with low expression (*P* < 0.05), blue indicates poorer survival in patients with low gene expression compared with those with high expression (*P* < 0.05) and gray indicates all other cases. Asterisk within the boxes denotes the *P* value from survival analysis using the R maxstat package (*P* ≥ 0.05: ns, *P < 0.05, **P < 0.01, ***P < 0.001, ****P < 0.0001). BRCA, breast invasive carcinoma; COAD, colon adenocarcinoma; LIHC, liver hepatocellular carcinoma; LUAD, lung adenocarcinoma.

**Fig. 3:**
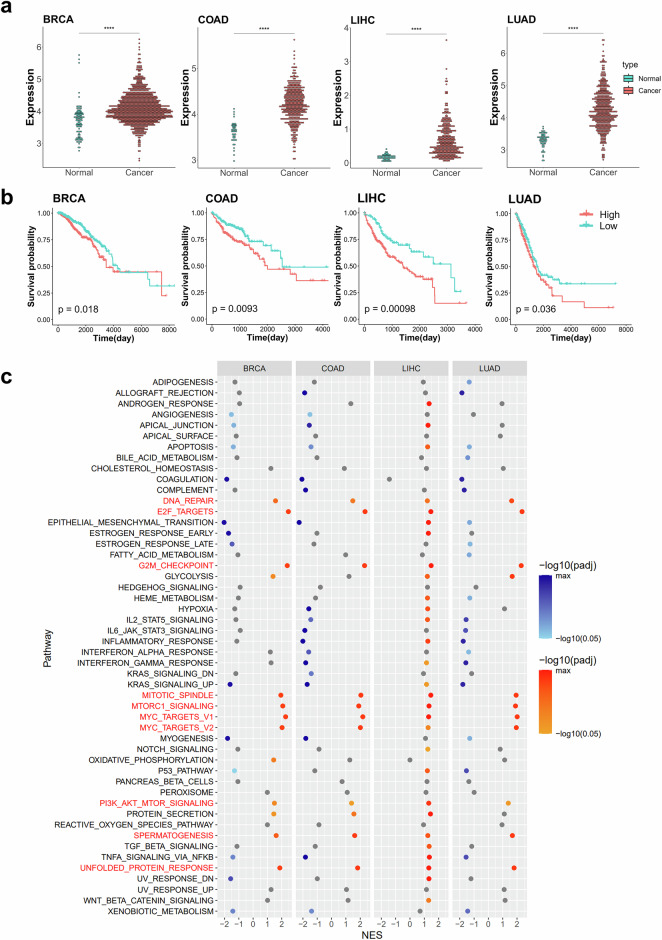
The expression level of the m^5^C writer, NSUN2, is highly associated with cell cycle progression. **a** Dotplots for NSUN2 gene expression in four cancer types. Expression values are in FPKM from the TCGA database. Red represents cancer tissues, and blue represents normal tissues. The asterisks above the plot represent the adjusted *P* value from a *t*-test (*P* ≥ 0.05: ns, *P < 0.05, **P < 0.01, ***P < 0.001, ****P < 0.0001). **b** Kaplan–Meier plots for overall survival based on NSUN2 gene expression from the TCGA database. The red and blue lines indicate patients with high and low expression levels of NSUN2, respectively. The *P* value is displayed within the graph, calculated using survival analysis from the R maxstat package. **c** Dotplots for GSEA results according to NSUN2 gene expression across four cancer types. GSEA was performed using the hallmark gene sets in the R fgsea package. Yellow represents pathways with a positive normalized enrichment score (NES) (enriched in patient with NSUN2 high expression) and a *P* value below 0.05, shown with a gradient according to the *P* value. Blue represents pathways with a negative normalized enrichment score (NES) (enriched in patient with NSUN2 low expression) and a *P* value below 0.05, also shown with a gradient. All other cases are shown in gray. The pathways commonly upregulated across the four cancer types are marked in red, while commonly downregulated pathways are marked in blue. BRCA, breast invasive carcinoma; COAD, colon adenocarcinoma; LIHC, liver hepatocellular carcinoma; LUAD, lung adenocarcinoma.

**Fig. 4:**
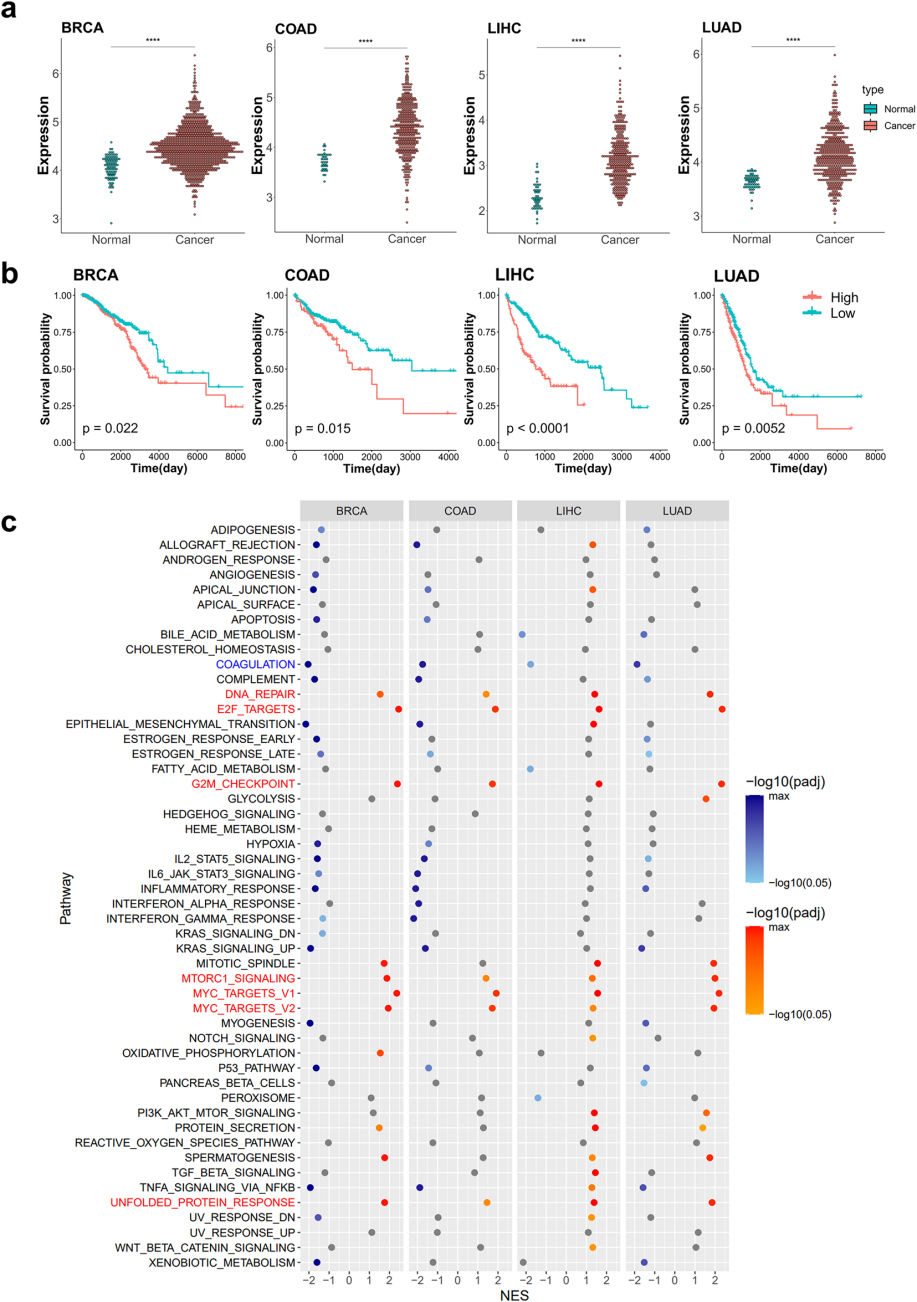
The expression level of the m^5^C writer, DNMT3B, is highly associated with cell cycle progression. **a** Dotplots for DNMT3B gene expression in four cancer types. The expression values are in FPKM from the TCGA database. Red represents cancer tissues, and blue represents normal tissues. The asterisks above the plot represent the adjusted *P* value from a *t*-test (*P* ≥ 0.05: ns, *P < 0.05, **P < 0.01, ***P < 0.001, ****P < 0.0001). **b** Kaplan–Meier plots for overall survival based on DNMT3B gene expression from the TCGA database. The red and blue lines indicate patients with high and low expression levels of DNMT3B, respectively. The *P* value is displayed within the graph, calculated using survival analysis from the R maxstat package. **c** The dotplots for GSEA results according to DNMT3B gene expression across four cancer types. A GSEA was performed using the hallmark gene sets in the R fgsea package. Yellow represents pathways with a positive normalized enrichment score (NES) (enriched in patients with high DNMT3B expression) and a *P* value below 0.05, shown with a gradient according to the *P* value. Blue represents pathways with a negative normalized enrichment score (NES) (enriched in patient with DNMT3B low expression) and a *P* value below 0.05, also shown with a gradient. All other cases are shown in gray. The pathways commonly upregulated across the four cancer types are marked in red, while commonly downregulated pathways are marked in blue. BRCA, breast invasive carcinoma; COAD, colon adenocarcinoma; LIHC, liver hepatocellular carcinoma; LUAD, lung adenocarcinoma.

**Fig. 5:**
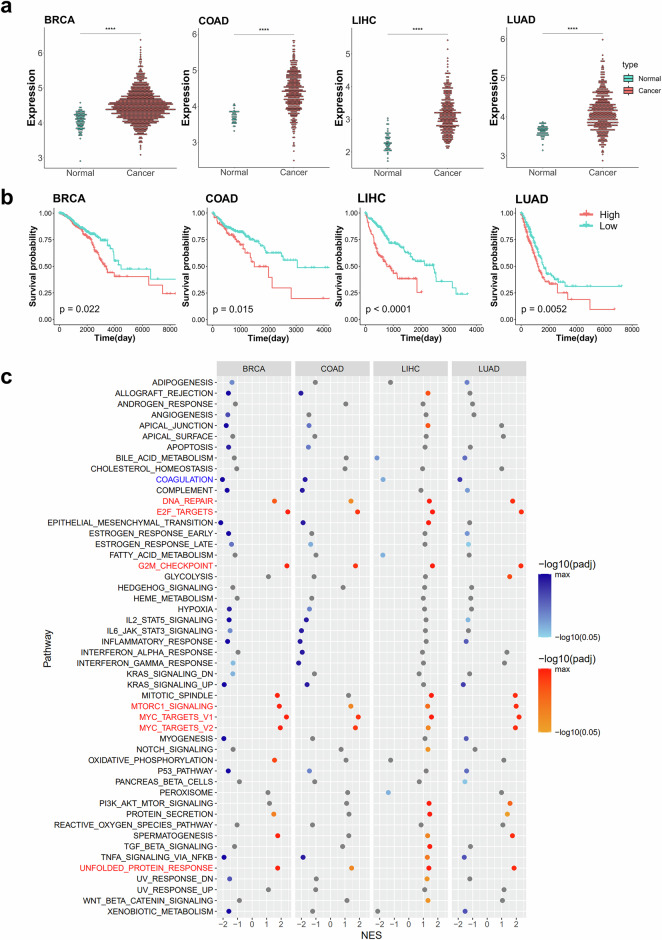
The expression level of the m^7^G reader, CBP20, is highly associated with cell cycle progression. **a** The dotplots for CBP20 gene expression in four cancer types. Expression values are in FPKM from the TCGA database. Red represents cancer tissues, and blue represents normal tissues. The asterisks above the plot represent the adjusted *P* value from a *t*-test (*P* ≥ 0.05: ns, *P < 0.05, **P < 0.01, ***P < 0.001, ****P < 0.0001). **b** Kaplan–Meier plots for overall survival based on CBP20 gene expression from the TCGA database. The red and blue lines indicate patients with high and low expression levels of CBP20, respectively. The *P* value is displayed within the graph, calculated using survival analysis from the R maxstat package. **c** Dotplots for GSEA results according to CBP20 gene expression across four cancer types. A GSEA was performed using the hallmark gene sets in the R fgsea package. Yellow represents pathways with a positive normalized enrichment score (NES) (enriched in patients with high CBP20 expression) and a *P* value below 0.05, shown with a gradient according to the *P* value. Blue represents pathways with a negative normalized enrichment score (NES) (enriched in patients with low CBP20 expression) and a *P* value below 0.05, also shown with a gradient. All other cases are shown in gray. The pathways commonly upregulated across the four cancer types are marked in red, while commonly downregulated pathways are marked in blue. BRCA, breast invasive carcinoma; COAD, colon adenocarcinoma; LIHC, liver hepatocellular carcinoma; LUAD, lung adenocarcinoma.

**Fig. 6:**
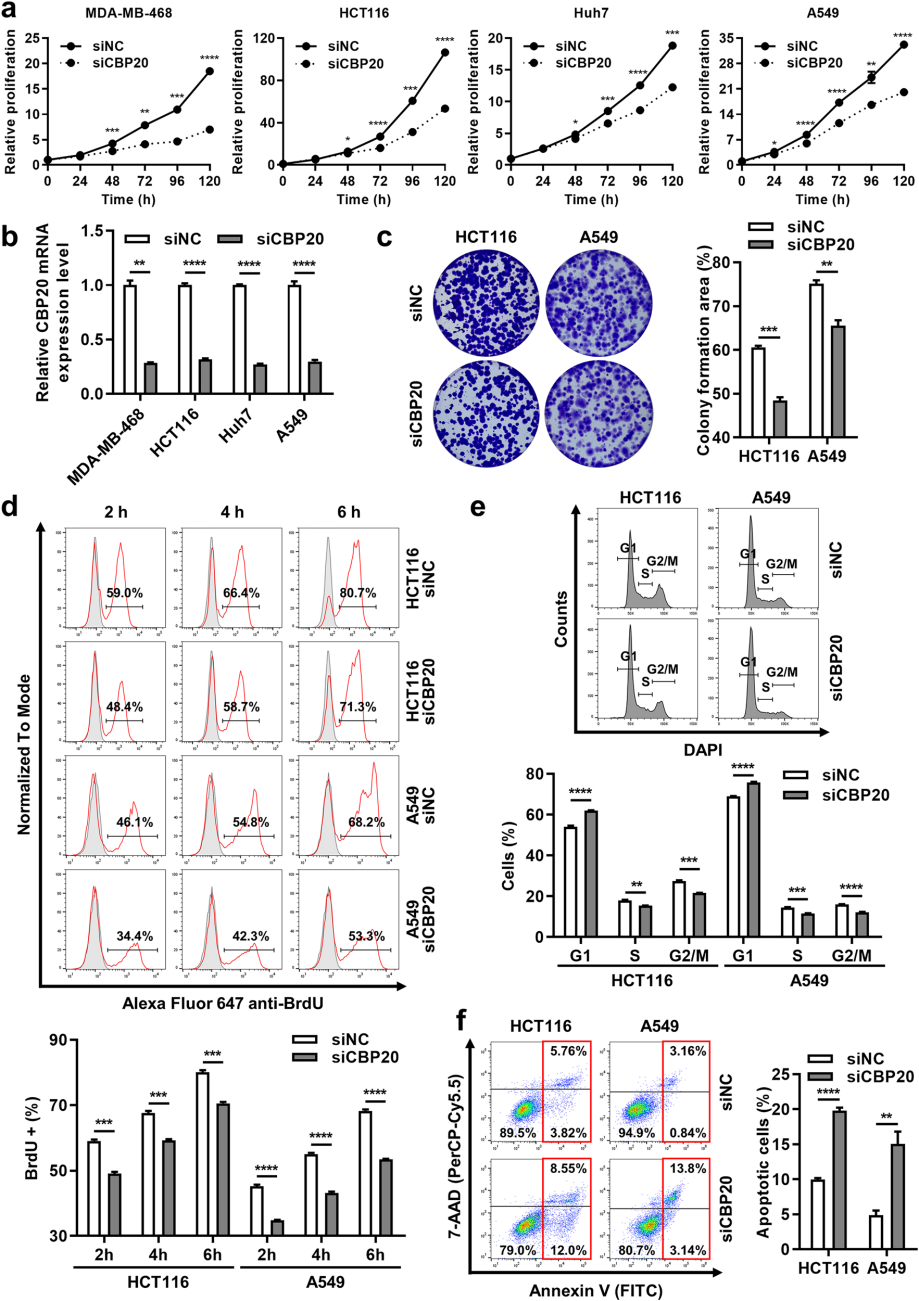
Depletion of CBP20 inhibits cell proliferation via G1 arrest in various cancer cells. **a** The proliferation of MDA-MB-468, HCT116, Huh7 and A549 cells after 96-well transfection with negative control siRNA (siNC) or siRNA for CBP20 (siCBP20) for indicated time points. The cell proliferation was determined using WST assays after transfection of siRNAs. **b** A validation of CBP20 knockdown by siRNAs in MDA-MB-468, HCT116, Huh7 and A549 cells. qRT–PCR was performed for CBP20 mRNA in four cell lines collected from cell proliferation assay at 48 h. **c** Colony formation of HCT116 and A549 cells after CBP20 depletion. The cells were reseeded 24 h after transfection and cultured for 14 days. The bar graph represents the average percentages of colony-formed area for each condition. **d** BrdU incorporation in CBP20-depleted HCT116 and A549 cells analyzed by flow cytometry. The gray lines indicate unstained samples for the control group, while the red lines indicate anti-BrdU stained samples for each condition. The bar graph shows the average percentages of BrdU incorporated cells at each time point. **e** A cell cycle analysis of HCT116 and A549 cells by flow cytometry after transfection of siRNAs. The bar graph presents the average percentages of each cell cycle phase in HCT116 and A549 cells. **f** Apoptosis analysis of HCT116 and A549 cells by flow cytometry after transfection of siRNAs. The apoptotic cells were assessed by Annexin V/7-AAD double staining, with the numbers in each graph representing the relative percentage of cells in each quadrant. The bar graph demonstrates average apoptotic cells including early and late apoptotic cells. All graphs are represented as mean ± standard deviation of experiments performed in triplicate. All *P* values were calculated by a *t*-test (*P* ≥ 0.05: ns, *P < 0.05, **P < 0.01, ***P < 0.001, ****P < 0.0001).

**Fig. 7:**
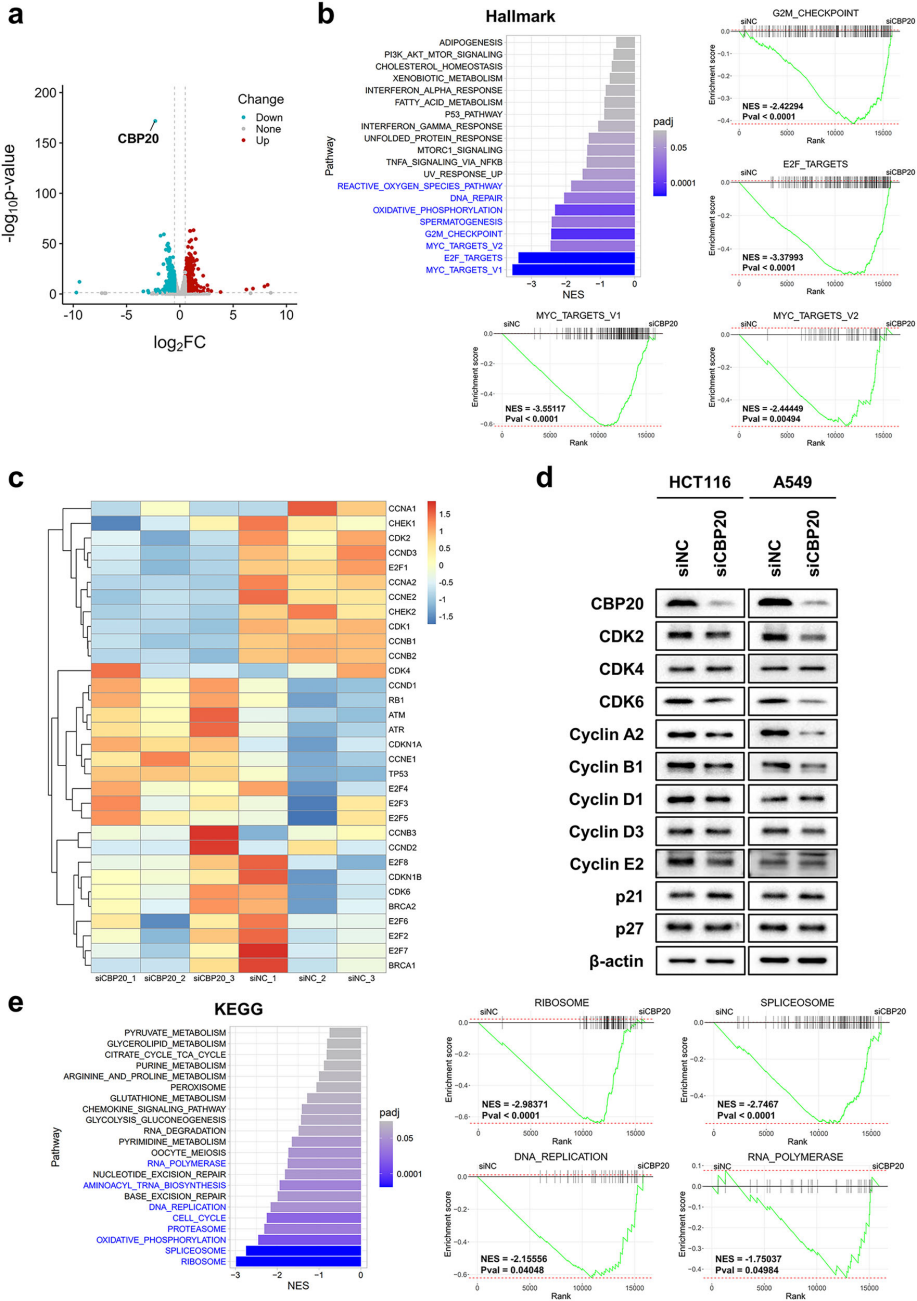
Transcriptomic analysis for cells with CBP20 depletion in lung cancer cells. **a** RNA sequencing (RNA-seq) analysis of A549 cells treated with negative control siRNA (siNC) or siRNA for CBP20 (siCBP20). RNA-seq data were analyzed using the R DESeq2 package and are presented as a volcano plot. The red dots represent genes upregulated with log_2_(fold change) >0.5 and *P* < 0.05, the blue dots represent genes downregulated under log_2_(fold change) <−0.5 and *P* < 0.05, and all other genes are shown as gray dots. **b** GSEA results from RNA-seq data using hallmark gene sets. Top left: GSEA was performed between siNC and siCBP20-treated cells, and significantly downregulated pathways (*P* < 0.05) are highlighted in blue. The GSEA enrichment plot shows the top four significantly downregulated pathways. Normalized enrichment score (NES) and *P* values are indicated next to the plots. The siNC group is located on the left side of the plot, and the siCBP20 group is on the right. **c** A heat map displaying the *Z*-score of genes involved in the cell cycle pathway using TPM values from RNA-seq data. The left three columns represent the siCBP20 group, and the right three columns represent the siNC group. The box colors indicate the *Z*-score. **d** Western blot of indicated proteins in HCT116 and A549 cells after transfection 48 h with siNC or siCBP20. β-actin was used as a loading control. **e** GSEA results from RNA-seq data using KEGG pathway gene sets. Left: GSEA was performed between siNC and siCBP20-treated cells and significantly downregulated pathways (*P* < 0.05) are marked in blue. Right: the GSEA enrichment plot displays four significantly downregulated pathways. The normalized enrichment score (NES) and *P* values are indicated next to the plots, with the siNC group (left) and the siCBP20 group (right) shown.

To elucidate the alterations in RNA modification machinery during carcinogenesis across different cancer types, we conducted a comparative analysis of RNA modification-related gene expression between normal and cancer tissues, utilizing datasets obtained from the TCGA database. We compared the expression difference of writer, eraser and reader genes involved in each of the nine types of RNA modification (gene list in Supplementary Table 1). The four cancer types chosen for our analysis are among those with notable global prevalence and substantial impact on mortality: breast cancer (BRCA), colorectal cancer (COAD), liver cancer (LIHC) and lung cancer (LUAD). Upon examining the overall trend, we noted that the expression levels of numerous genes linked to RNA modification generally exhibit an increase in cancer tissues compared with normal tissues across nearly all types of modification (Fig. 1a–i). Especially in the cases of LIHC, nearly all genes exhibit a substantial increase in expression to a noteworthy degree. Among these genes, we identified several genes whose expression was significantly heightened across cancer tissues, irrespective of cancer type (*P* < 0.05): (1) TRMT6, TRMT10C and TRMT61A for m^1^A writer, ALKBH1 for m^1^A eraser, and YTHDF1 and YTHDF2 for m^1^A reader (Fig. 1a); (2) NSUN1, NSUN2, NSUN4, NSUN5, DNMT1, DNMT3A and DNMT3B for m^5^C writer, ALKBH1 for m^5^C eraser, and ALYREF, FMRP and YTHDF2 for m^5^C reader (Fig. 1b); (3) METTL1, WRD4, WBSCR22, TRM112 and BCDIN3D for m^7^G writer, TGS1 for m^7^G eraser, and eIF4E, CBP20 and CBP80 for m^7^G reader (Fig. 1d); (4) DKC1, PUS1, PUS7, PUS7L, TRUB1, TRUB2, RPUSD1, RPUSD2 and RPUSD3 for pseudouridine writer (Fig. 1e); (5) TUT1 for uridylation writer, LSM2, LSM4, LSM5 and LSM7 for uridylation reader (Fig. 1f); (6) CMRT1 for 2′-*O*-Me writer (Fig. 1g); (7) NAT10 for ac^4^C writer (Fig. 1h); and (8) ADAR1 for the adenosine-to-inosine editing writer (Fig. 1i). These data indicate a substantial alteration in RNA modifications throughout the process of carcinogenesis.

### Prognostic effect of gene expression of RNA modification writers, erasers and readers across cancer types

Next, we investigated whether alterations in the expression of genes related to RNA modification correlate with patient survival outcomes. We performed survival data analysis comparing patients with high and low expression levels of each gene across four types of cancer. Interestingly, while several genes exhibited high expression levels in cancer tissues (Fig. 1a–i), the prognostic effect of this elevated expression varied across different cancer types (Fig. 2a–i). For example, patients with elevated expression of the m^5^C writer NSUN4 experienced poorer prognoses in BRCA and LIHC but showed improved prognosis in COAD and LUAD (Fig. 2b). Similarly, patients with heightened expression of the m^5^C reader ALYREF had poorer prognosis in LIHC and LUAD but exhibited better prognosis in BRCA and COAD (Fig. 2b). In the overall trend, in LIHC, most genes demonstrated reduced survival rates when their expression levels were high (Fig. 2a–i). On the contrary, patients with COAD exhibited relatively lower survival rates in a larger proportion of genes when exhibiting lower expression levels (Fig. 2a–i). Among the RNA modification-related genes examined, only three genes were identified to have a detrimental effect on survival across all four types of cancer when their expression was high in cancer tissues: NSUN2 and DNMT3B, writer genes for m^5^C and CBP20, a reader gene for m^7^G (Fig. 2b, d). Since these three genes exhibited elevated expression levels in the cancer tissues of all four cancer types examined, we proceeded to conduct further analysis on their functions.

### The expression levels of RNA modification-related NSUN2, DNMT3B, and CBP20 are highly associated with cell cycle progression

To understand the molecular functions of RNA modification-related genes, NSUN2, DNMT3B, and CBP20, we performed a GSEA comparing patients with high and low expression levels of each gene. The initial analysis focuses on NSUN2, which serves as the writer gene for m^5^C. The expression of NSUN2 markedly increased in cancer tissues (Fig. 3a) and patients with high expression of NSUN2 demonstrated poorer overall survival across four distinct cancer types (Fig. 3b). The GSEA, based on expression levels categorized into high and low groups from survival analysis within each cancer type, unveiled several pathways commonly enriched across the four cancer types. Notably, NSUN2 high-expression group exhibited significant enrichment in cell-cycle-related pathways, including ‘E2F targets’, ‘G2M checkpoint’, ‘Mitotic spindle’, and ‘Myc targets’ (Fig. 3c). In addition, pathways related with ‘DNA repair’, ‘MTORC1 signaling’, ‘PI3K-AKT-MTOR signaling’ and ‘Unfolded protein response’, all linked to cell proliferation, were also enriched in NSUN2 high-expression group (Fig. 3c). While no pathways consistently showed enrichment in the NSUN2 low-expression group across all four cancer types, ‘Apoptosis’ gene set was enriched in the NSUN2 low-expression group in three cancer types (BRCA, COAD, and LUAD), alongside enrichment of immune-related pathways and the p53 pathways in specific cancers (Fig. 3c). These data suggest the potential tumor-promoting roles of NSUN2.

Next, we investigated the molecular functions of DNMT3B, the writer gene for m^5^C. The expression of DNMT3B exhibited a significant increase in cancer tissues (Fig. 4a), with patients displaying high DNMT3B expression demonstrating poorer overall survival across four distinct cancer types (Fig. 4b). Similar to NSUN2, patients with DNMT3B high-expression group demonstrated significant enrichment in cell-cycle-related pathways, including ‘E2F targets’, ‘Myc targets’, ‘G2M checkpoint’ and ‘Mitotic spindle’, and proliferation-related gene sets, including ‘DNA repair’, ‘PI3K-AKT-MTOR signaling’ and ‘Unfolded protein response’ (Fig. 4c). In addition, ‘Apoptosis’ gene set was enriched in patients with DNMT3B low expression in three cancer types (BRCA, COAD and LUAD) (Fig. 4c).

We also analyzed the molecular functions of CBP20, the reader gene for m^7^G. The expression of CBP20 showed a notable increase in cancer tissues (Fig. 5a), correlating with poorer overall survival in patients with high CBP20 expression across four distinct cancer types (Fig. 5b). GSEA demonstrated that patients exhibiting high expression of CBP20 showed significant enrichment in cell-cycle-related pathways, such as ‘E2F targets’, ‘Myc targets’ and ‘G2M checkpoint’, as well as in proliferation-related gene sets including ‘DNA repair’, ‘MTORC1 signaling’ and ‘Unfolded protein response’ (Fig. 5c). Moreover, genes in ‘Coagulation’ gene set were significantly enriched in the CBP20 low-expression group (Fig. 5c). Taken together, this data analysis suggests that all three RNA modification-related genes (NSUN2, DNMT3B and CBP20) are associated with cell cycle regulation and cell proliferation.

### Knockdown of CBP20 inhibits cell proliferation via G1 arrest in various cancer cells

To figure out whether RNA modification-related genes actually impact the overall cancer-related phenotypes, we used four types of cancer cell line for a loss of function study. Among the three genes previously described, NSUN2 and DNMT3B are already reported for their oncogenic roles in various cancer types. NSUN2 enhances cell proliferation, migration and invasion through diverse mechanisms in several types of cancer, including breast, colorectal, lung, liver, gastric, pancreatic cancer and osteosarcoma^26–33^. Consistently, depletion of NSUN2 inhibited cell proliferation in four types of cancer cell (Supplementary Fig. 1a,b). DNMT3B influences oncogenic phenotypes in NSCLC and colorectal cancer through regulation by specific miRNAs^34,35^. In addition, DNMT3B impacts breast, gastric, prostate, cervical and bladder cancers by modulating the methylation of target genes^36–40^. Indeed, reduced levels of DNMT3B suppressed cell growth in three cancer cell lines, with the exception of the breast cancer cell line (Supplementary Fig. 1c,d). Therefore, we focused on CBP20 to elucidate its functional role in cancer development and progression. In four types of cancer cell, depletion of CBP20 significantly decreased cell proliferation (Fig. 6a, b). In addition, knockdown of CBP20 reduced colony formation abilities in four cancer cell lines (Fig. 6c and Supplementary Fig. 2a). To determine whether the effects of CBP20 knockdown on cell proliferation are attributable to reduced cell growth or increased cell death, we examined BrdU incorporation. Incorporations of BrdU, a newly synthesized DNA marker^41^, were markedly diminished with CBP20 knockdown in colorectal, liver, and lung cancer cells at all time points (Fig. 6d and Supplementary Fig. 2b). In addition, CBP20 inhibition substantially increased G1 arrest in these three cell lines (Fig. 6e and Supplementary Fig. 2c). Upon analyzing apoptotic cell death, we found that about 90% of the cells remained viable in control. However, depletion of CBP20 led to an increase in apoptotic cell death in breast, colorectal and lung cancer cells (Fig. 6f and Supplementary Fig. 2d). Consequently, the depletion of CBP20 leads to decreased cell proliferation primarily through G1 arrest, with additional induction of apoptosis.

We also investigated the role of RNA m^7^G modification in mediating the effects of CBP20. Depletion of the m^7^G writers, METTL1 and RNMT, resulted in antiproliferative effects across all four cancer cell lines (Supplementary Fig. 3a–d), suggesting that the m^7^G writers, METTL1 and RNMT, are both linked to cell proliferation. CBP20 has two conserved tyrosine residues (Tyr^20^ and Tyr^43^) that position m^7^G between them^42–44^. To verify the role of CBP20 in RNA modification, we utilized plasmids encoding wild-type CBP20 and a m^7^G-binding-deficient mutant form (Y20A and Y43A) (Supplementary Fig. 4a). The reduced BrdU incorporation observed upon CBP20 knockdown was restored by overexpression of wild-type CBP20, whereas overexpression of the m^7^G-binding-deficient mutant failed to restore this phenotype (Supplementary Fig. 4b), indicating that the effects observed upon CBP20 knockdown are attributable to the specific loss of its m^7^G-binding function, rather than a general disruption of RNA biology caused by the absence of CBP20. Moreover, to mimic m^7^G depletion, we stratified patients with cancer based on the expression levels of m^7^G writer genes and conducted a survival analysis for those in the lower half of m^7^G writer gene expression. Even among patients with low m^7^G writer gene expression, those with high CBP20 expression generally exhibited poor prognosis, with a few exceptions (Supplementary Fig. 5a–g). This may be attributed to the presence of multiple m^7^G methylation-related genes in cancer cells, where the decreased expression of a single m^7^G writer gene may have a limited impact on CBP20 function in patients.

### RNA sequencing reveals downregulation of cell-cycle-related pathways after CBP20 depletion

To investigate the molecular mechanisms underlying CBP20-induced cell cycle arrest in cancer cells, we performed RNA sequencing after CBP20 knockdown in all four types of cancer cell (Fig. 7a and Supplementary Fig. 6a). We found that 1170 genes are significantly upregulated, and 600 genes are significantly downregulated followed by CBP20 knockdown (adjusted *P* < 0.05 and |log_2_(fold change)| >0.5) in lung cancer cells (Fig. 7a). We conducted GSEA using the hallmark gene sets to gain an overview of global gene expression changes. The results showed a general downregulation of cell-cycle-related pathways in CBP20-knockdown samples of lung and colorectal cancer cells, including ‘E2F targets’, ‘G2M checkpoint’ and ‘Myc targets’, consistent with previous findings from the analysis of the TCGA public database (Figs. 5c and 7b and Supplementary Fig. 6b). Moreover, CBP20 depletion led to the downregulation of ‘Myc targets’ in liver cancer cells and ‘Interferon response’ in breast cancer cells (Supplementary Fig. 6b). Specifically, we investigated the expression of genes involved in cyclins (CDK1, CDK2, CDK4, CDK6, CCNA1-2, CCNB1-3, CCND1-3 and CCNE1-2)^45,46^, regulatory proteins (TP53, RB1, CDKN1A, CDKN1B and E2F1-8)^47–49^ and checkpoint regulators (BRCA1-2, CHEK1-2, ATM and ATR)^50,51^. CBP20 knockdown induced widespread changes in the expression of cell-cycle-related genes (Fig. 7c and Supplementary Fig. 6c), and we identified a general downregulation of CDK1, CDK2, CCNA1-2, CCNB1-2, CCND3 and CCNE2 in the CBP20-knockdown samples of lung cancer cells (Fig. 7c). Moreover, we observed an increased mRNA expression of regulatory proteins such as RB1, TP53, ATM and ATR in lung cancer cells (Fig. 7c). To validate the results of RNA sequencing analysis, we examined protein levels following siRNA-mediated knockdown of CBP20. The results showed reduced expression of CDK2, CDK6, cyclin B1 and cyclin E2 across all four cell lines, along with decreased expression of CDK4, cyclin A2, cyclin D1 and cyclin D3 in specific cell lines. By contrast, p21 expression was increased in all four cell lines, and the p27 levels were elevated specifically in the breast cancer cells (Fig. 7d and Supplementary Fig. 7a). In addition, in specific cell lines, the mRNA levels of CDK2, CDK6, CCNB1, CCND3 and CCNE2 were decreased, while the expression of CDKN1A was increased (Supplementary Fig. 7b). Considering both mRNA and protein expression changes, the observed CBP20 depletion-mediated downregulation of CDKs and a broad range of cyclins may lead to inhibition of the G1–S and G2–M transition^52^. The increased expression of RB1, TP53, ATM and ATR is likely to further induce cell cycle arrest and activate DNA damage response, slowing down cell cycle progression^53,54^. In addition, the upregulation of p21 could inhibit the interaction between CDK2 and cyclin E, thus preventing the G1–S transition^55^. As a result, cells treated with siRNA targeting CBP20 exhibited slower cell cycle progression, leading to reduced cell proliferation and survival, as previously observed (Fig. 6). To explore the regulatory targets of CBP20 at the mRNA level, we performed RNA immunoprecipitation (RIP)-sequencing using a CBP20 antibody (Supplementary Fig. 8a), and a total of 227 genes were identified through peak calling using bioinformatic tools (Supplementary Fig. 8b). These genes were significantly enriched in cell-cycle-related pathways (*P* < 0.05), such as ‘G2M checkpoint’, ‘Mitotic spindle’, ‘E2F targets’ and ‘Myc targets’ in hallmark gene sets, ‘Cell cycle’ in Kyoto Encyclopedia of Genes and Genomes (KEGG) gene sets and ‘Cell cycle process’ and ‘Cell cycle’ in Gene Ontology Biological Processes gene sets (Supplementary Fig. 8c).

To further explore the impact of CBP20 depletion on cellular metabolism and function, we conducted additional GSEA using the KEGG gene set. The analysis revealed a marked decrease in pathways involved in RNA metabolism-related functions, including ‘Ribosome’, ‘Spliceosome’, ‘DNA replication’ and ‘RNA polymerase’, following CBP20 knockdown (Fig. 7e and Supplementary Fig. 6d). Another notable finding was the overall decrease in m^7^G writers and uridylation readers, along with a general increase in m^5^C erasers in the siCBP20 group (Supplementary Fig. 9a–i). Moreover, alterations in the expression of various other RNA modification-related genes were observed, further supporting the hypothesis that CBP20 may have a critical role in regulating gene expression.

### Raloxifene, purpurogallin and enoxacin decrease viability of cancer cells and mimic the effects of CBP20 depletion on cell cycle markers

**Fig. 8:**
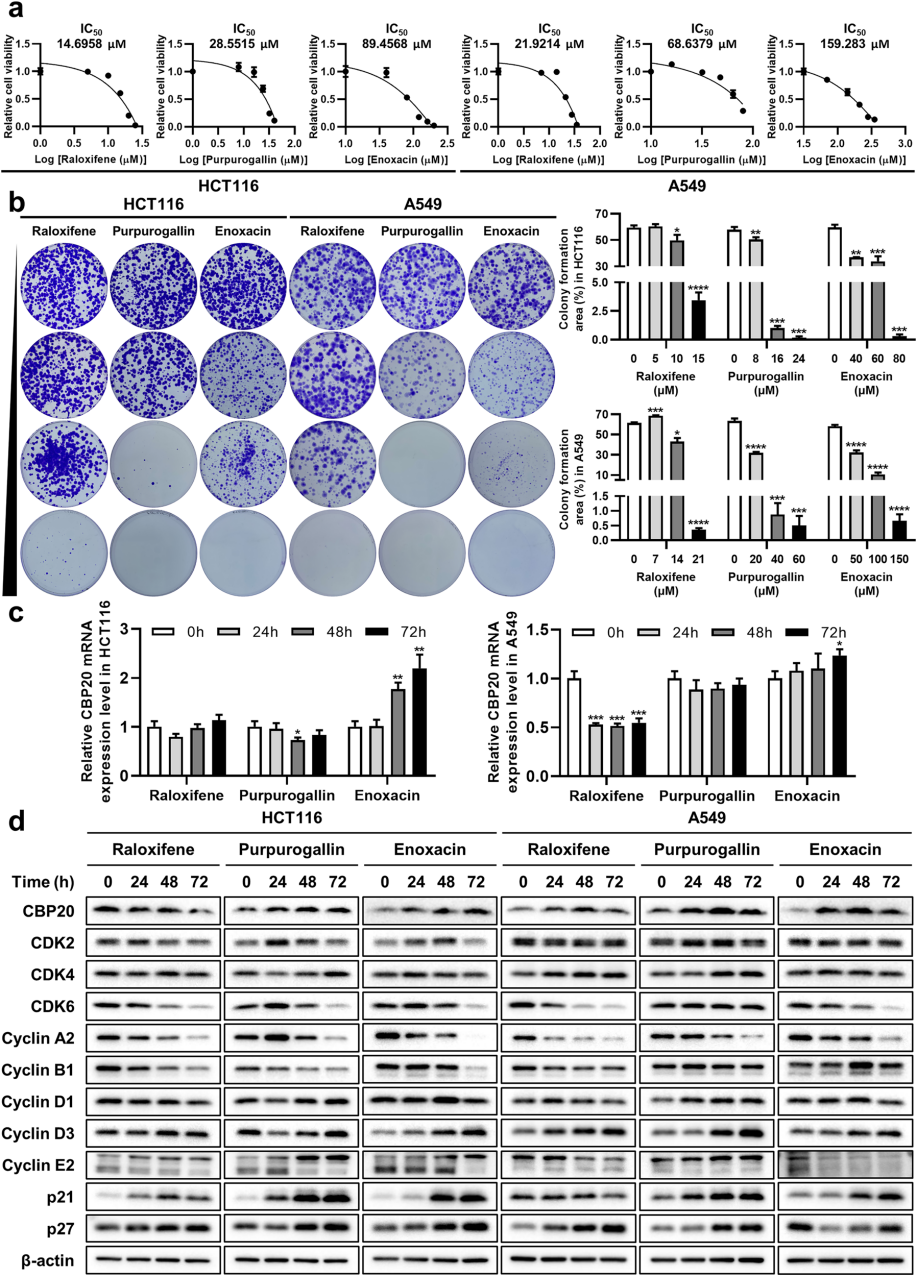
Effects of CBP20 depletion-mimicking chemicals on cancer cell proliferation and cell cycle markers. **a** The effect of raloxifene, purpurogallin or enoxacin on cancer cell viability. Cytotoxicity for each drug measured by WST after exposing the cells to the drug for 72 h in HCT116 and A549 cells. The IC_50_ values were calculated using CompuSyn. **b** The colony formation of HCT116 and A549 cells treating with indicated concentrations of raloxifene, purpurogallin or enoxacin for 14 days. The bar graphs represent the average percentages of colony-formed area for each drug condition. **c** The effect of raloxifene, purpurogallin or enoxacin on CBP20 mRNA expression. qRT–PCR was performed for CBP20 mRNA in HCT116 and A549 cells after treating raloxifene (HCT116, 15 μM; A549, 20 μM), purpurogallin (HCT116, 25 μM; A549, 60 μM) or enoxacin (HCT116, 80 μM; A549, 150 μM) for indicated time points. **d** The effect of raloxifene, purpurogallin or enoxacin on CBP20 and cell-cycle-related proteins. A western blot of indicated proteins in HCT116 and A549 cells was performed after treating raloxifene (HCT116, 15 μM; A549, 20 μM), purpurogallin (HCT116, 25 μM; A549, 60 μM) or enoxacin (HCT116, 80 μM; A549, 150 μM) for indicated time points. All graphs are represented as mean ± standard deviation. All *P* values were calculated by a *t*-test (*P* ≥ 0.05: ns, *P < 0.05:, **P < 0.01, ***P < 0.001, ****P < 0.0001).

Given the lack of known drugs targeting CBP20, we employed SigCom LINCS database^24^ to screen for compounds with similar effects to CBP20 depletion. From the RNA sequencing analysis of lung cancer cells, we identified 225 DEGs using the criteria of the adjusted *P* < 0.05 and |log_2_(fold change)| >1, resulting in 157 upregulated genes (log_2_(fold change) >1) and 68 downregulated genes (log_2_(fold change) <−1) (Supplementary Table 2). These DEGs were used as input data for SigCom LINCS, which led to the selection of raloxifene, purpurogallin and enoxacin for testing their effects on cancer (Supplementary Table 3). Although these drugs are not primarily used in cancer, we finally chose them because some studies have reported their anticancer effects^56–63^. When treated to cancer cell lines, all three drugs reduced cell viability in both colorectal and lung cancer cells (Fig. 8a). Colorectal cancer cells showed greater sensitivity to drug reactivity compared with lung cancer cells in all three drugs, with raloxifene being the most promising of the three candidates considering half maximal inhibitory concentration, IC_50_ values (Fig. 8a). In CBP20-depleted cells, we observed little difference in drug sensitivities based on CBP20 expression levels (Supplementary Fig. 10a), probably due to the incomplete depletion of CBP20 by siRNA-mediated knockdown (Supplementary Fig. 10b). It is possible that the inhibition of residual CBP20 by each of the three drugs still contributed to the suppression of tumor cell growth or that these drugs exerted anticancer effects through CBP20-independent mechanisms. Colony formation assays at sub-IC_50_ concentrations showed a reduction in colony formation with increasing drug concentrations in both cancer cell lines (Fig. 8b). To evaluate the similarity of their effects to CBP20 knockdown, we analyzed mRNA and protein levels following drug treatment and assessed the impact of the drugs on CBP20 and cell-cycle-related gene expression. CBP20 mRNA expression decreased only with raloxifene treatment in lung cancer cells and increased with enoxacin in both cell lines, while protein levels decreased only in colorectal cancer cells treated with raloxifene and appeared to increase in other conditions (Fig. 8c, d). Thus, the effect of all three drugs is not associated with a reduction in CBP20 expression. However, the changes in cell cycle markers under most conditions resembled the effects of CBP20 depletion, with reductions in CDK2, CDK6, cyclin A2 and cyclin B1 levels, along with increased p21 levels. In addition, in contrast to the knockdown experiments, these drugs elevated the levels of CDK inhibitor, p27 (Fig. 7c and Fig. 8d). Considering the alterations in cell cycle markers, we suggest that raloxifene, purpurogallin and enoxacin mimic the effects of CBP20 depletion without directly inhibiting CBP20 expression.

To determine whether the effects of CBP20 depletion or CBP20 depletion-mimicking drugs are cancer cell-specific, we performed CBP20 knockdown or drug treatment in noncancerous cell lines using normal colonocyte (CCD-18Co) and normal lung fibroblast cells (MRC-5 and WI-38). Our results showed that CBP20 depletion inhibited cell proliferation in all three tested normal cell lines (Supplementary Fig. 11a,b). These data suggest the possibility that CBP20 may cause comparable damage to normal tissues, underscoring the importance of evaluating its potential adverse effects in future studies. However, given that CBP20 mRNA expression is significantly elevated in cancer tissues compared to normal tissues (Fig. 1d) and its protein expression is higher in cancer cell lines than in normal cell lines (Supplementary Fig. 11c), targeting CBP20 in cancer may exert a more pronounced effect on cancer cells. Consistent with this hypothesis, treatment of normal cell lines with CBP20 depletion-mimicking drugs resulted in higher IC_50_ values, particularly for purpurogallin and enoxacin, compared with cancer cell lines (Supplementary Fig. 11d,e). Therefore, although the safety window needs to be carefully considered, targeting CBP20 holds potential as a viable therapeutic strategy for cancer treatment.

### In silico docking analysis of CBP20/CBP80 complex with CBP20 depletion-mimicking drugs

To investigate whether the drug candidates inhibit CBP20 function, we performed in silico docking analysis of CBP20 with raloxifene, purpurogallin and enoxacin. Since CBP20 forms a heterodimer with CBP80 to constitute the cap-binding complex (CBC)^42^, we assessed the binding affinities of these drugs to the CBP20/CBP80 complex rather than CBP20 alone. The results predicted that all three CBP20 depletion-mimicking drugs bind at the interface between CBP20 and CBP80 (Supplementary Fig. 12a–f). The estimated binding affinities were 86.7 nM for raloxifene, 274.7 nM for purpurogallin and 276.0 nM for enoxacin (Supplementary Fig. 12g), which correlate closely with the observed IC_50_ values from the cell viability assay (Fig. 8a). These findings raise the possibility that candidate drugs could exert their inhibitory effects on CBP20 by altering its interaction with CBP80 exerted. However, this possibility requires experimental validation to confirm the exact mechanism of action.

## Discussion

Cancer cells undergo extensive genomic alterations, often accompanied by complex remodeling of gene expression across multiple regulatory layers. These dysregulated processes contribute to aberrant signaling pathways and cellular dysfunctions that drive tumor progression, metastasis and resistance to therapy. While previous studies have primarily focused on genes or proteins, recent advancements in experimental techniques^64,65^ and the discovery of noncoding RNAs^66^ have highlighted the significant role of post-transcriptional regulation in gene expression. RNA modifications have gained increasing attention due to their critical role in post-transcriptional regulation, contributing substantially to the characteristics of cancer cells. Notably, m^6^A modification of RNAs influences RNA stability, splicing, translation and degradation, thereby contributing to cancer development through diverse processes in colorectal cancer^67,68^, breast cancer^69^ and other types of cancer^70,71^. However, the precise roles of various other types of RNA modification in cancer biology have not been fully elucidated. Therefore, our study was initiated to provide a more comprehensive investigation into the role of RNA modifications across cancer types.

m^5^C is one of the most commonly found RNA modifications. Previous studies have revealed that m^5^C is linked to various RNA forms, leading to alterations in RNA metabolism and contributing to cancer migration, invasion, metastasis and other cellular processes^72^. Among the factors that regulate m^5^C, NSUN2, the m^5^C writer, has been reported to be upregulated and negatively impact survival in gastric cancer^30^. The involvement of NSUN2 has been linked to c-Myc in gallbladder carcinoma^73^. This finding is consistent with our data, which demonstrated the enrichment of c-Myc target pathways associated with high NSUN2 expression across all four cancer types (BRCA, COAD, LIHC and LUAD). In addition to c-Myc, our data also indicated significant activity in cell-cycle-related pathways. This observation is further corroborated by in vitro studies suggesting that NSUN2 influences cell-cycle-related factors in osteosarcoma^74^. Another m^5^C writer, DNMT3B, exhibited high expression in four types of cancer tissue in our analysis, which was associated with poor survival outcomes. Moreover, existing studies have reported elevated DNMT3B expression in breast, liver, lung, and ovarian cancer cells, as well as in the majority of TCGA datasets^75,76^. A notable observation was the trend of reduced immune-related pathways associated with the upregulation of both NSUN2 and DNMT3B. There are reports indicating that overexpression of NSUN2 in human kidney cells promotes cancer development by suppressing the immune system via glucose metabolism^77^. Similarly, overexpression of DNMT3B in keratinocytes has been associated with a reduction in immune-related pathways^78^. In colorectal cancer, it has also been reported that lower m^5^C scores correlate with increased immune cell infiltration^79^.

The RNA modification m^7^G is frequently found on mRNA and tRNA, where it modulates RNA stability and the efficiency of translation^80,81^. Previous studies have mainly focused on the METTL1/WDR4 m^7^G writer complex, which has been found to be highly expressed in various cancers and linked to proteins involved in cell cycle regulation and proliferation^82–84^. Our analysis also confirmed a significant upregulation of METTL1 and WDR4 in cancer tissues compared to normal tissues (Fig. 1d). However, study on CBP20 (NCBP2), the reader protein for m^7^G, remains limited, which we specifically targeted in this study. In oral squamous cell carcinoma (OSCC), CBP20 expression is elevated in cancer tissues, and higher levels were linked to a more than two fold increase in the hazard ratio for survival^85^. In pancreatic cancer, a higher CBP20 expression was associated with reduced survival rates, and in vivo studies demonstrated a decrease in tumor size upon CBP20 knockdown using shRNA^86^. Additional research in colon cancer cells revealed that CBP20 expression is elevated compared with normal intestinal cells, with pathway analysis indicating enrichment in cell-cycle-related pathways^87^. In *Drosophila*, targeted depletion of CBP20 among the 3q29 genes resulted in widespread dysregulation of cell cycle pathways and apoptosis, leading to developmental abnormalities^88^, which suggests that CBP20 plays a key role in promoting cell cycle progression and inhibiting apoptosis. Overall, these findings indicate that targeting CBP20 may consistently impede cancer progression and proliferation across multiple cancer types, in agreement with our findings and pointing to a more widespread pattern.

In addition, elevated CBP20 expression is correlated with a marked reduction in immune-related pathways in our GSEA data, except for liver cancer (Fig. 5c). In head and neck cancer, decreased CBP20 expression has been linked to enhanced immune-mediated tumor attacks^89^. By contrast, in liver cancer, higher CBP20 levels have been reported to be positively associated with immune cell infiltration^90^. These findings highlight the need for further investigation into the role of CBP20 in immune regulation across various cancers.

CBP20 is a key component of the cap-binding complex (CBC), which consists of CBP20 and CBP80. RNA guanine-7 methyltransferase (RNMT), in complex with its activating partner RNMT-activating mini-protein (RAM), primarily catalyzes the m^7^G modification of the mRNA cap structure. CBC binds to 5′ cap of mRNA and plays a crucial role in multiple gene expression regulatory processes, including transcription, splicing, RNA 3′ end processing, RNA export and translation^91,92^. Our findings suggest that CBP20 regulates the expression of cell cycle regulators at both the mRNA and protein levels through post-transcriptional mechanisms. Previous studies have indicated that the m^7^G structure of mRNAs is a critical regulatory point for cell-cycle-related genes. Elevated CBP20 expression has been shown to activate the c-JUN/MEK/ERK pathway in an m7G-dependent manner, leading to increased transcription of CCND1^86,93,94^. The m^7^G-binding protein eIF4E has been reported to enhance the nucleocytoplasmic transport and translation of cell-cycle-related genes, including cyclin D1 and c-Myc^95^. Furthermore, one of the m7G readers, eIF3d, has been reported to regulate the translation of CDK1, cyclin A, cyclin B, p21 and p53^96^, highlighting the critical role of m^7^G in controlling the expression of cell cycle regulators. Also, siRNA-mediated knockdown of CBP80 led to the misregulation of approximately 400 genes, suggesting that CBC functions in both common and gene-specific regulatory pathways^97^. In our RNA sequencing analysis, we found around 1,770 DEGs following CBP20 depletion (Fig. 7a). Notably, several MYC and E2F target genes, which are key regulators of the cell cycle, were significantly downregulated upon CBP20 depletion (Fig. 7b), indicating its role in regulating specific gene expression. Moreover, in our RIP-sequencing analysis, 227 genes were identified as targets of CBP20, and these genes were enriched in cell-cycle-related pathways (Supplementary Fig. 8c). However, it remains unclear whether CBC broadly influences gene expression or primarily regulates a specific subset of genes^92^. Further investigation is required to elucidate the precise molecular mechanisms by which CBP20 specifically enhances the expression of cell-cycle-related genes.

In this study, we identified several drug candidates, including raloxifene, purpurogallin and enoxacin, which mimic the effects of CBP20 depletion in cancer cells through transcriptome-based drug repositioning. These compounds demonstrated anticancer activity in cancer cell culture models by modulating various cell-cycle-related factors (Fig. 8). Raloxifene is a selective estrogen receptor modulator used for postmenopausal osteoporosis treatment and has reduced breast cancer incidence significantly^98^. Raloxifene has been shown to induce apoptosis by directly interacting with the aryl hydrocarbon receptor (AhR) in ER-negative hepatoma cells^56^. In addition, it suppressed S-phase progression in vascular smooth muscle cells stimulated by PDGF by preventing the phosphorylation of Rb, which is normally hyperphosphorylated during the G1–S transition^57^. Purpurogallin, a natural compound derived from the oxidation of pyrogallol^99^, has demonstrated anticancer activity by inhibiting DNA synthesis^58^ and EGFR activity^59^. It disrupts polo-box domain binding of PLK1, a key regulator of cell cycle stages including mitosis^60,61^. In addition, purpurogallin directly interacts with MEK1/2, attenuating the ERK signaling pathway, which suppresses cell proliferation, reduces levels of cyclin A2 and cyclin B1, and activates PARP, leading to cell cycle arrest and apoptosis^62^. Enoxacin, a fluoroquinolone with potent antibacterial activity, targets both Gram-negative and Gram-positive bacteria by inhibiting bacterial DNA gyrase and topoisomerase IV^100^. In addition to its antibacterial properties, enoxacin enhances the production of tumor-suppressive miRNAs by interacting with the miRNA biosynthesis protein TAR RNA-binding protein 2 (TRBP)^63^. Moreover, LZ-106, an analog of enoxacin, has been found to inhibit cell growth, induce apoptosis, and arrest the cell cycle in the G1 phase by generating p53-dependent intracellular ROS and modulating cell cycle regulators such as CDK4, CDK6 and p27^101^. Collectively, these findings suggest that these compounds exhibit cytotoxicity and modulate cell cycle marker expression through diverse molecular mechanisms.

The in silico docking analysis conducted using Pharmaco-Net^25^ provided molecular-level insights into how CBP20 depletion-mimicking compounds may influence CBP20/CBP80 functionality and contribute to the observed phenotypic effects. The binding of raloxifene, purpurogallin and enoxacin to CBP20/CBP80 complex, as predicted by the AI-Dock and DeepCalici-plus module on Pharmaco-Net, suggests that these compounds may exert CBP20 inhibition by changing its interaction with CBP80, potentially making CBP20 unstable. However, the minimal impact of CBP20 knockdown on drug sensitivity (Supplementary Fig. 10a,b) also raised the possibility that the drug effects may not be mediated by CBP20 inhibition. Therefore, the precise molecular mechanisms of the candidate drugs remain unclear and require additional experimental validation.

## Supplementary information

Supplementary Information

Supplementary Tables

## Data Availability

The main article and supplemental materials contain all the data related to this investigation.
