# Supplementary material for: Comprehensive profiling of RNA modification-related genes identifies RNA m7G binding protein CBP20 as a therapeutic target for tumor growth inhibition
Source: Exp Mol Med. 2025 Sep 1;57(9):1978–95. doi: 10.1038/s12276-025-01531-z (PMC12508125; doi:10.1038/s12276-025-01531-z)
Supplement: Supplementary file 1 — Supplementary Information [file 12276_2025_1531_MOESM1_ESM.pdf]

Supplementary Fig. 1

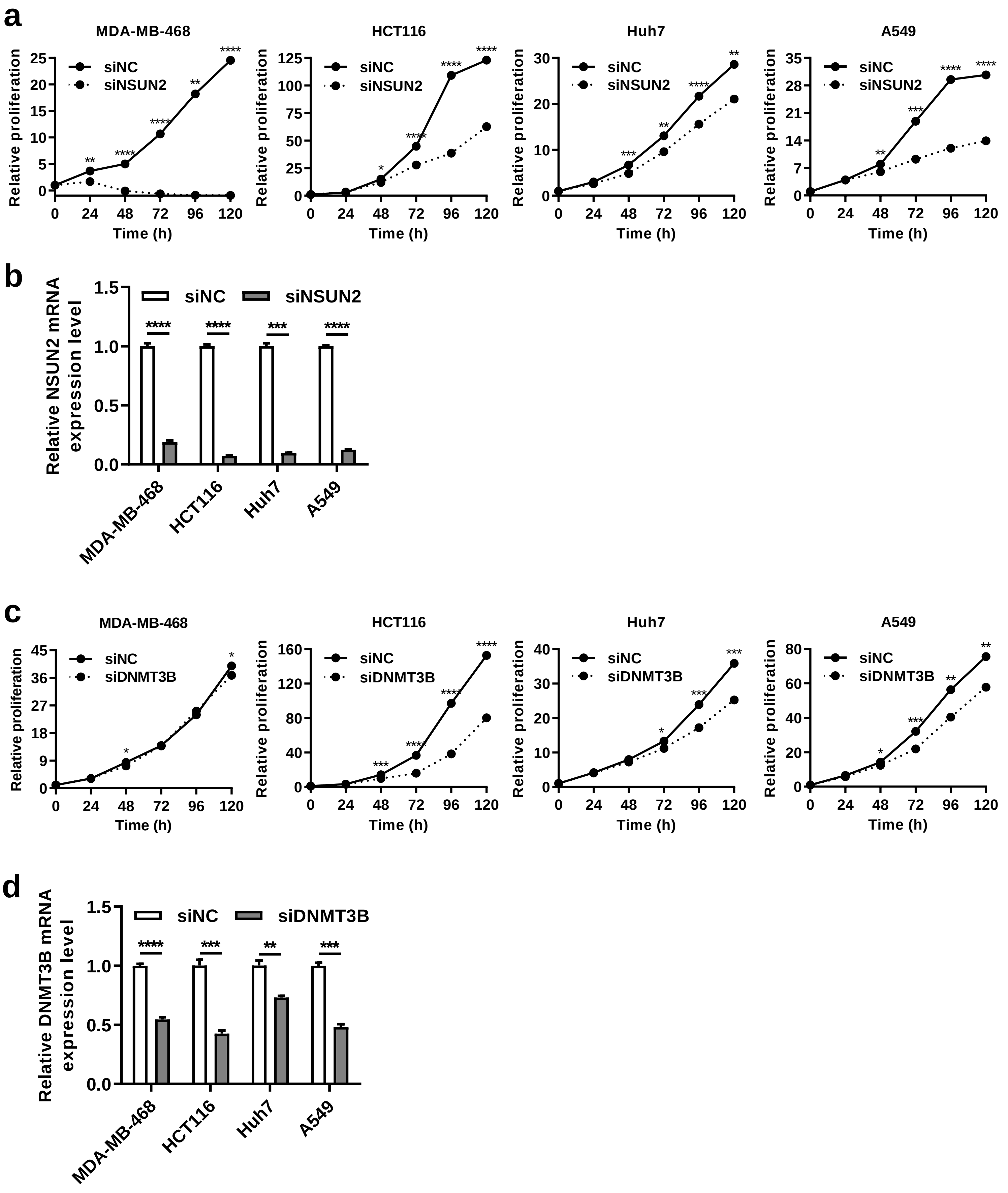

Supplementary Fig. 1 Depletion of NSUN2 and DNMT3B inhibits cell proliferation in various cancer cells.

a Proliferation of MDA-MB-468, HCT116, Huh7, and A549 cells after 96-well transfection with negative control siRNA (siNC) or siRNA for NSUN2 (siNSUN2) for indicated time points. Cell proliferation was determined using WST assays after transfection of siRNAs.

b Validation of NSUN2 knockdown by siRNAs in MDA-MB-468, HCT116, Huh7, and A549 cells. Quantitative real-time PCR (qRT-PCR) was performed for NSUN2 mRNA in four cell lines harvested from cell proliferation assay at 48 hours.

c Proliferation of MDA-MB-468, HCT116, Huh7, and A549 cells after 96-well transfection with negative control siRNA (siNC) or siRNA for DNMT3B (siDNMT3B) for indicated time points. Cell proliferation was determined using WST assays after transfection of siRNAs.

d Validation of DNMT3B knockdown by siRNAs in MDA-MB-468, HCT116, Huh7, and A549 cells. Quantitative real-time PCR (qRT-PCR) was performed for DNMT3B mRNA in four cell lines harvested from cell proliferation assay at 48 hours.

All graphs are represented as mean ± SD of experiments performed in triplicate. All p values were calculated by t-test ($p \geq 0.05$: ns, $p < 0.05$: *, $p < 0.01$: **, $p < 0.001$: ***, $p < 0.0001$: ****).

Supplementary Fig. 2

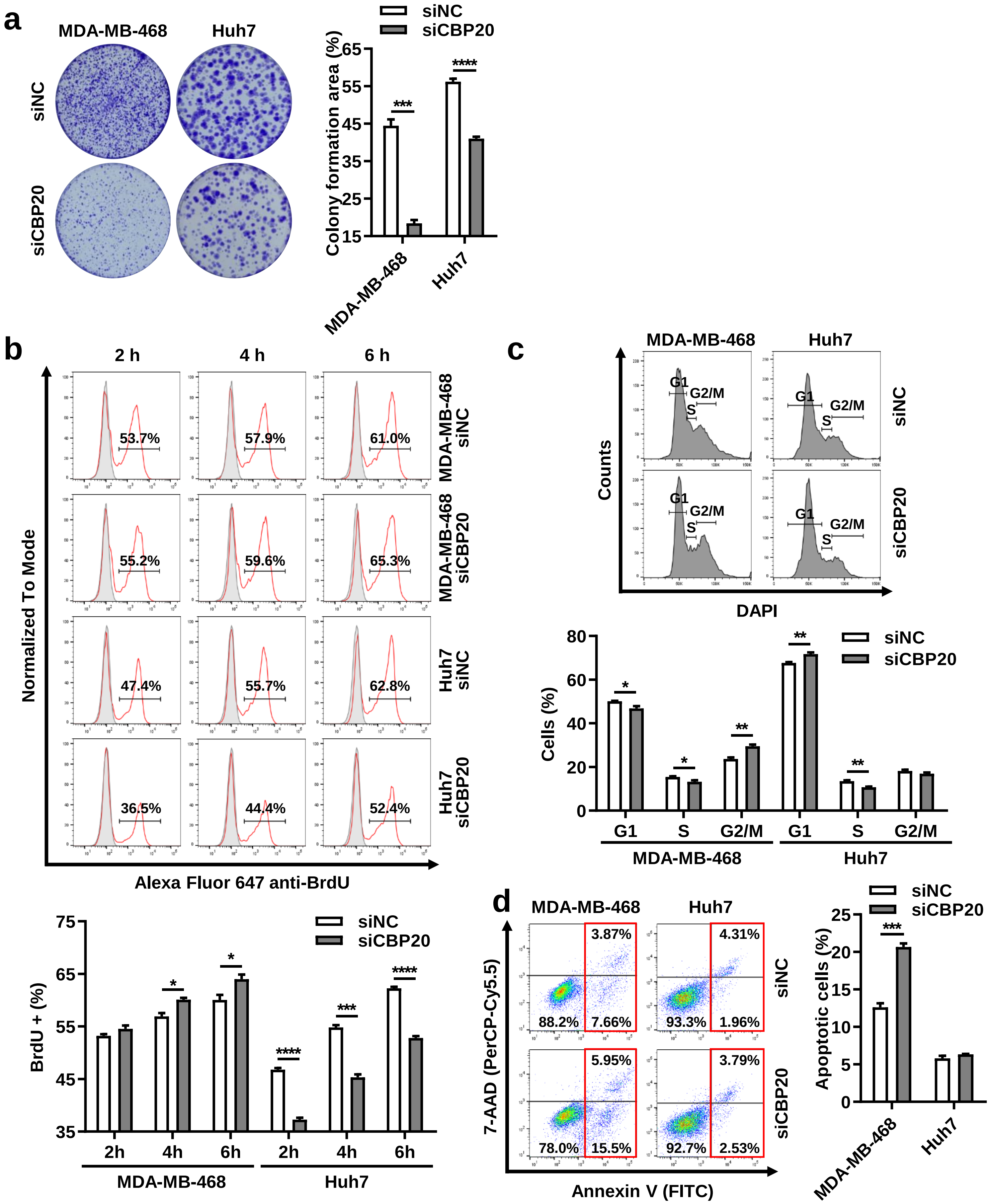

Supplementary Fig. 2 Depletion of CBP20 inhibits cell proliferation by reducing BrdU incorporation or increasing apoptosis.

a Colony formation of MDA-MB-468 and Huh7 cells after CBP20 depletion. Cells were reseeded 24 hours after transfection and cultured for 14 days. The bar graph represents the average percentages of colony-formed area for each condition.

b Bromodeoxyuridine (BrdU) incorporation in CBP20-depleted MDA-MB-468 and Huh7 cells analyzed by flow cytometry. Gray lines indicate unstained samples for the control group, while red lines indicate anti-BrdU stained samples for each condition. The bar graph shows the average percentages of BrdU incorporated cells at each time point.

c Cell cycle analysis of MDA-MB-468 and Huh7 cells by flow cytometry after transfection of siRNAs. The bar graph presents the average percentages of each cell cycle phase in MDA-MB-468 and Huh7 cells.

d Apoptosis analysis of MDA-MB-468 and Huh7 cells by flow cytometry after transfection of siRNAs. Apoptotic cells were assessed by Annexin V/7-AAD double staining, with the numbers in each graph representing the relative percentage of cells in each quadrant. The bar graph demonstrates average apoptotic cells including early and late apoptotic cells.

All graphs are represented as mean ± SD of experiments performed in triplicate. All p values were calculated by t-test ($p \geq 0.05$: ns, $p < 0.05$: *, $p < 0.01$: **, $p < 0.001$: ***, $p < 0.0001$: ****).

Supplementary Fig. 3

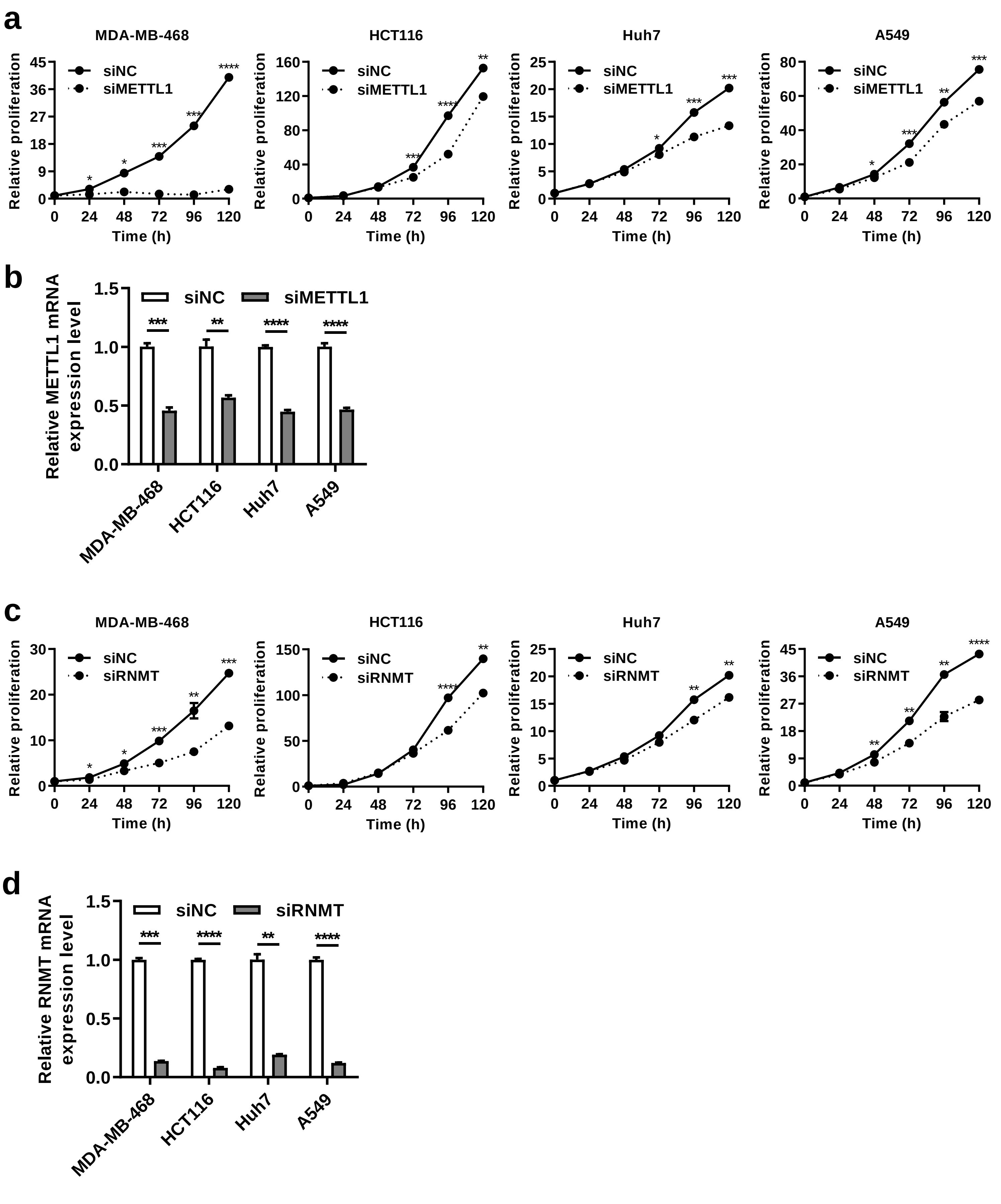

Supplementary Fig. 3 Depletion of METTL1 and RNMT inhibits cell proliferation in various cancer cells.

a Proliferation of MDA-MB-468, HCT116, Huh7, and A549 cells after 96-well transfection with negative control siRNA (siNC) or siRNA for METTL1 (siMETTL1) for indicated time points. Cell proliferation was determined using WST assays after transfection of siRNAs.

b Validation of METTL1 knockdown by siRNAs in MDA-MB-468, HCT116, Huh7, and A549 cells. Quantitative real-time PCR (qRT-PCR) was performed for METTL1 mRNA in four cell lines harvested from cell proliferation assay at 48 hours.

c Proliferation of MDA-MB-468, HCT116, Huh7, and A549 cells after 96-well transfection with negative control siRNA (siNC) or siRNA for RNMT (siRNMT) for indicated time points. Cell proliferation was determined using WST assays after transfection of siRNAs.

d Validation of RNMT knockdown by siRNAs in MDA-MB-468, HCT116, Huh7, and A549 cells. Quantitative real-time PCR (qRT-PCR) was performed for RNMT mRNA in four cell lines harvested from cell proliferation assay at 48 hours.

All graphs are represented as mean ± SD of experiments performed in triplicate. All p values were calculated by t-test ($p \geq 0.05$: ns, $p < 0.05$: *, $p < 0.01$: **, $p < 0.001$: ***, $p < 0.0001$: ****).

Supplementary Fig. 4

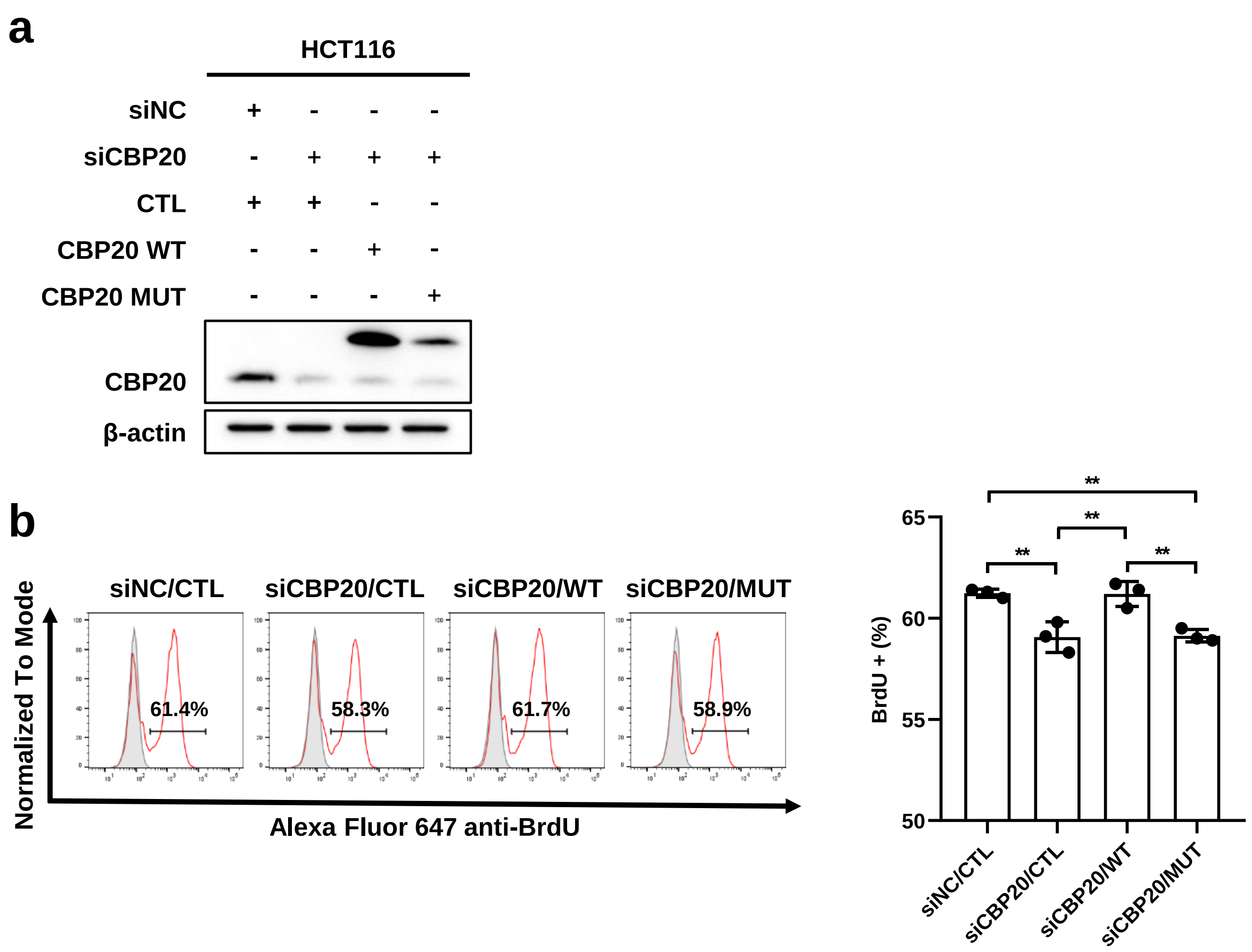

Supplementary Fig. 4 CBP20 enhanced BrdU incorporation in an m7G-dependent manner.

a Western blot of CBP20 protein in HCT116 cells after transfection 48 hours with siRNA (siNC or siCBP20) and plasmid (CTL; control vector, CBP20 WT, or CBP20 MUT). β-actin was used as a loading control.

b Bromodeoxyuridine (BrdU) incorporation in HCT116 cells with indicated condition analyzed by flow cytometry. Gray lines indicate unstained samples for the control group, while red lines indicate anti-BrdU stained samples for each condition. The bar graph shows the average percentages of BrdU incorporated cells for 2 hours. A graph is represented as mean ± SD of experiments performed in triplicate. All p values were calculated by one-way ANOVA multiple comparisons ($p \geq 0.05$: ns, $p < 0.05$: *, $p < 0.01$: **, $p < 0.001$: ***, $p < 0.0001$: ****).

Supplementary Fig. 5

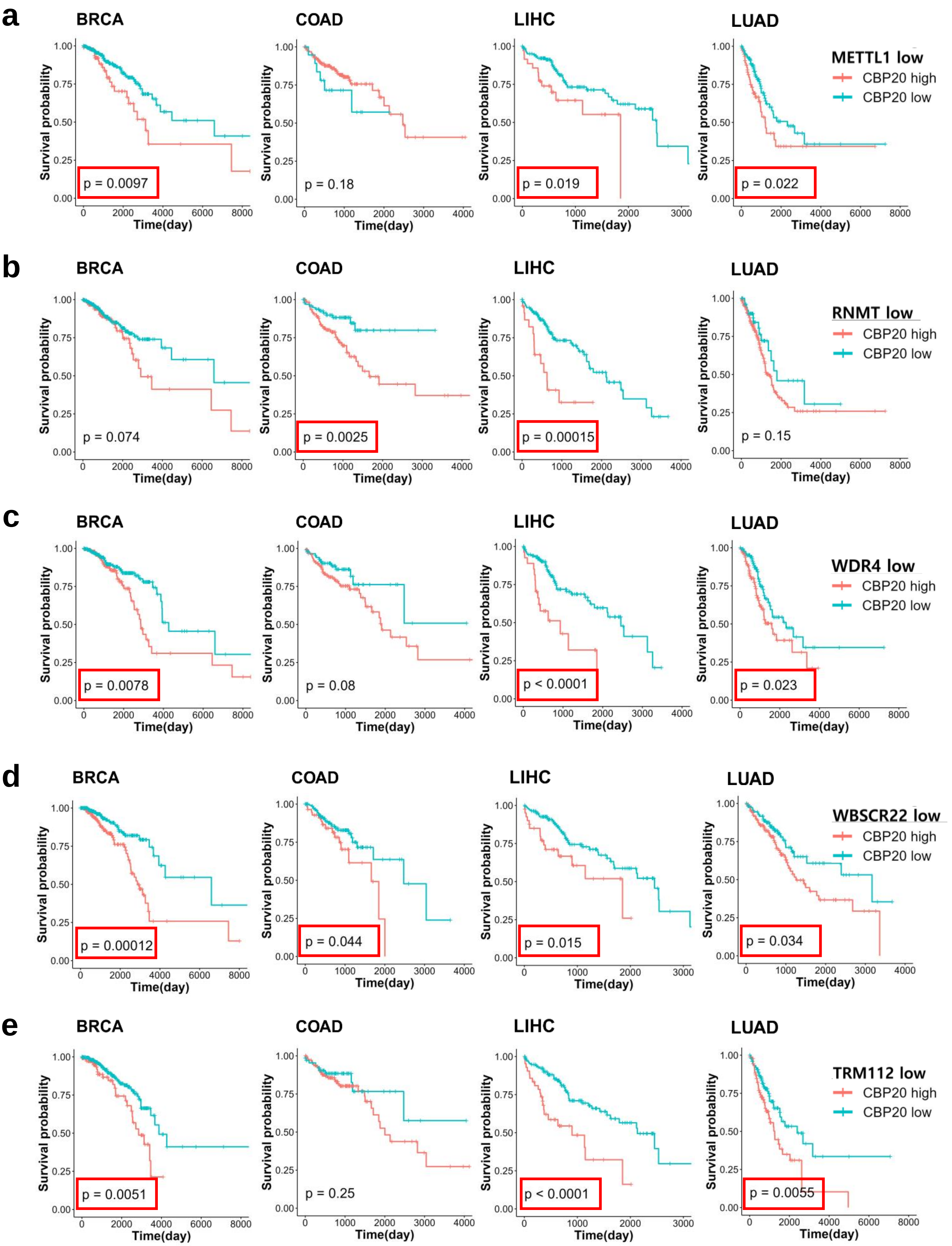

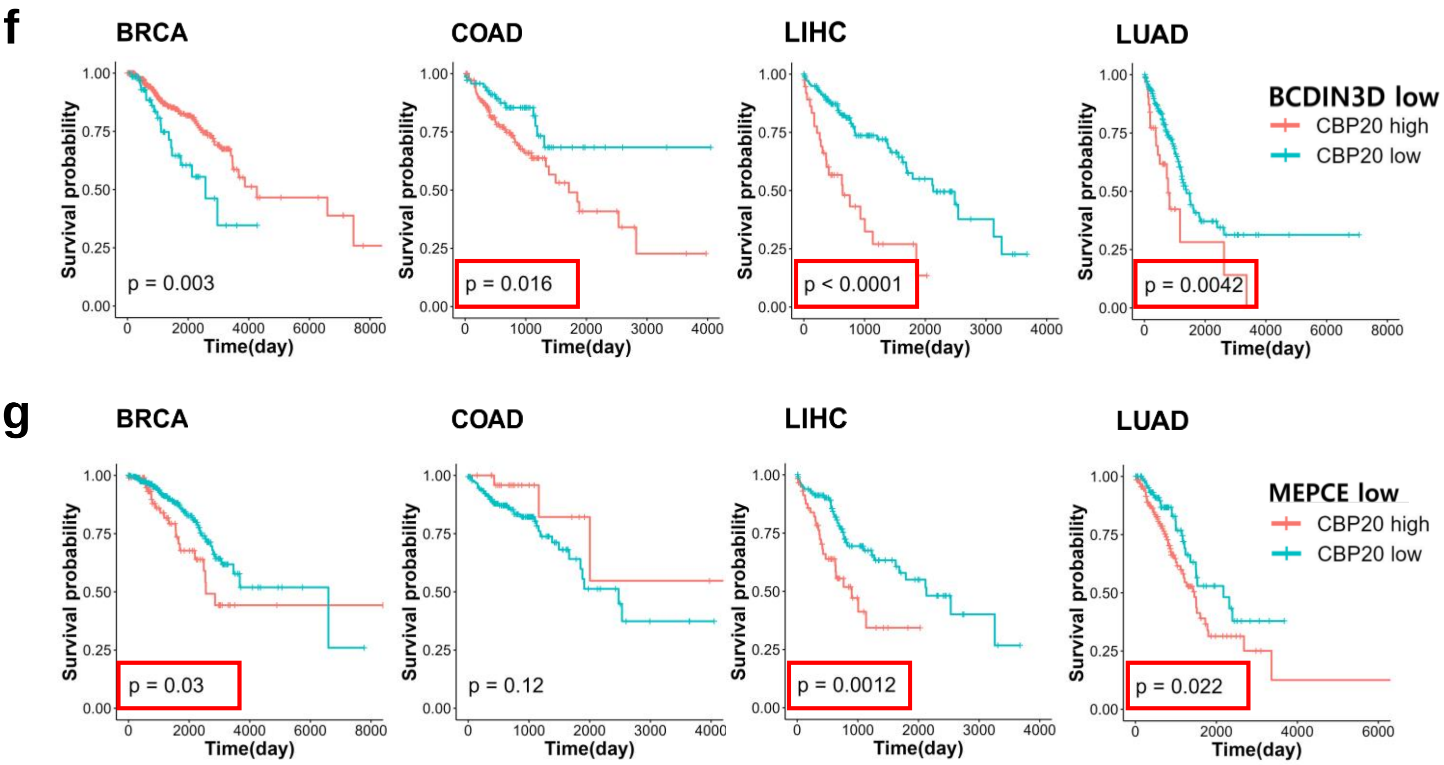

Supplementary Fig. 5 High CBP20 expression generally exhibited poor prognosis among patients with low m7G writer gene expression.

a-g Kaplan-Meier plots for overall survival based on m7G writer gene expression from the TCGA database. Red and blue lines indicate patients with high and low expression levels of CBP20, respectively. The p-value is displayed within the graph, calculated using survival analysis from the R maxstat package. The survival plots are categorized as follows: METTL1 low (a); RNMT low (b); WDR4 low (c); WBSCR22 low (d); TRM112 low (e); BCDIN3D low (f); MEPCE low (g). The red boxes indicate statistically significant p-values, suggesting that patients with high CBP20 expression exhibited poorer prognosis. BRCA, breast invasive carcinoma; COAD, colon adenocarcinoma; LIHC, liver hepatocellular carcinoma; and LUAD, lung adenocarcinoma.

Supplementary Fig. 6

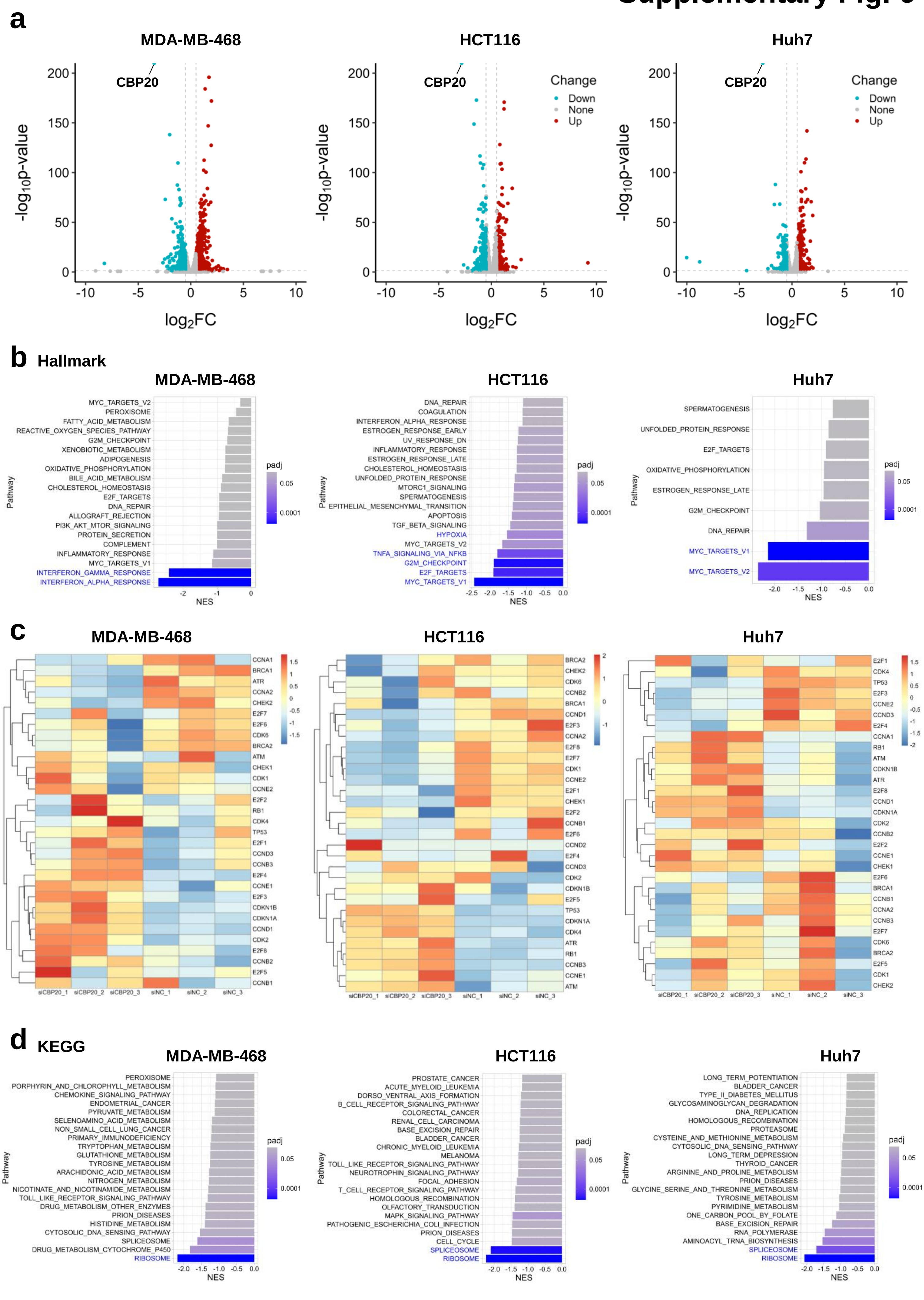

Supplementary Fig. 6 Transcriptomic analysis for cells with CBP20 depletion in MDA-MB-468, HCT116, and Huh7 cells.

a RNA sequencing (RNA-seq) analysis of MDA-MB-468, HCT116, and Huh7 cells treated with negative control siRNA (siNC) or siRNA for CBP20 (siCBP20). RNA-seq data were analyzed using the R DESeq2 package and are presented as a volcano plot. Red dots represent genes upregulated with $\log_2$[Fold change] > 0.5 and $p < 0.05$, blue dots represent genes downregulated under $\log_2$[Fold change] < -0.5 and $p < 0.05$, and all other genes are shown as grey dots.

b GSEA results from RNA-seq data using Hallmark gene sets. GSEA was performed between siNC and siCBP20-treated cells, and significantly downregulated pathways ($p < 0.05$) are highlighted in blue.

c Heatmap displaying Z-score of genes involved in the cell cycle pathway using TPM values from RNA-seq data. The left three columns represent the siCBP20 group, and the right three columns represent the siNC group. Box colors indicate the Z-score.

d GSEA results from RNA-seq data using KEGG pathway gene sets. GSEA was performed between siNC and siCBP20-treated cells, and significantly downregulated pathways ($p < 0.05$) are marked in blue.

Supplementary Fig. 7

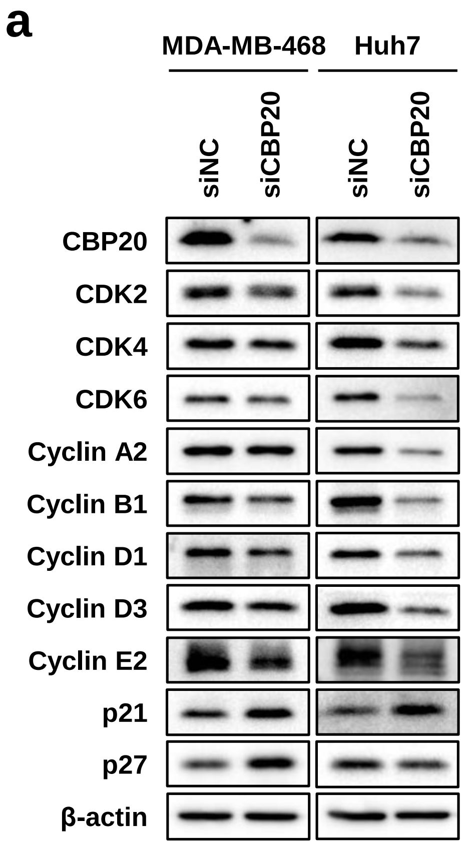

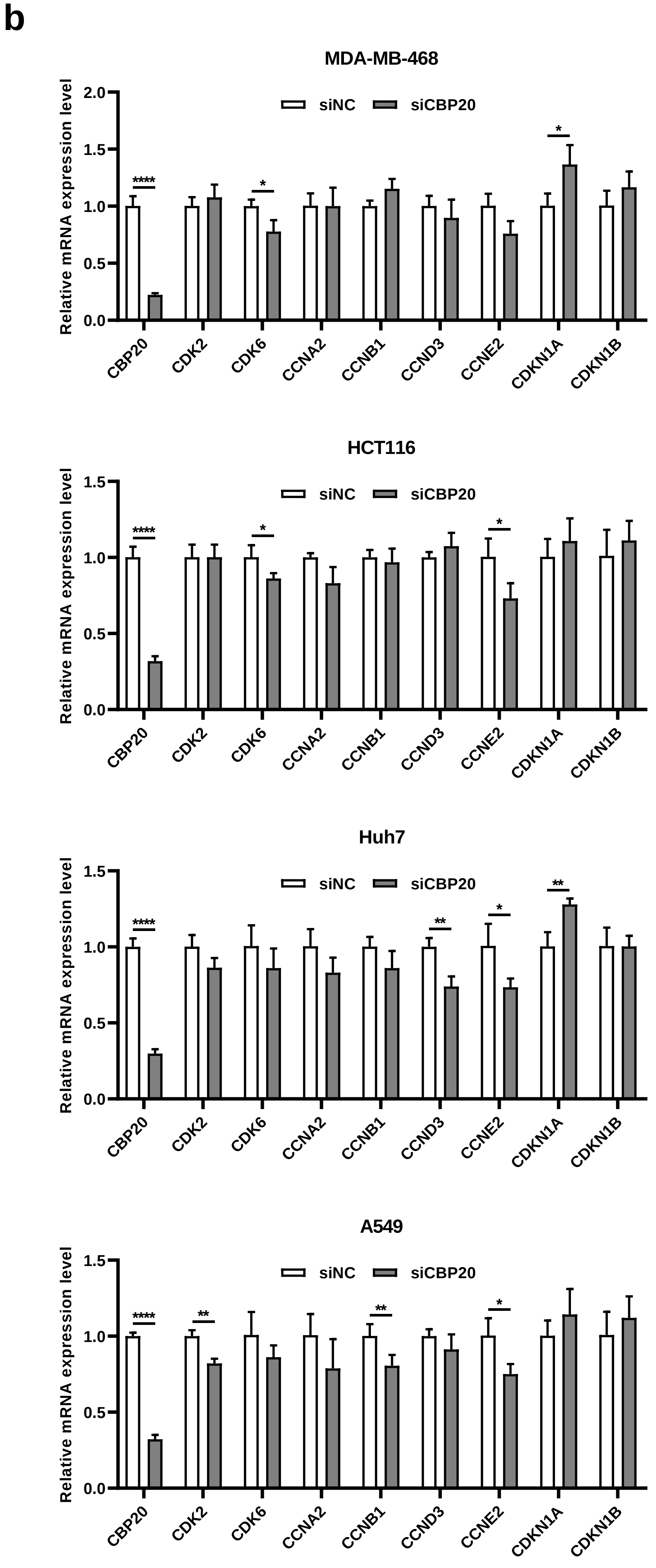

Supplementary Fig. 7 Depletion of CBP20 affects expression levels of proteins and mRNAs related to cell cycle.

a Western blot of indicated proteins in MDA-MB-468 and Huh7 cells after transfection 48 hours with siNC or siCBP20. β-actin was used as a loading control.

b Quantitative real-time PCR (qRT-PCR) was performed for indicated mRNAs in four cell lines after transfection 48 hours with siNC or siCBP20.

All graphs are represented as mean ± SD of experiments performed in triplicate. All p values were calculated by t-test ($p \geq 0.05$: ns, $p < 0.05$: *, $p < 0.01$: **, $p < 0.001$: ***, $p < 0.0001$: ****).

Supplementary Fig. 8

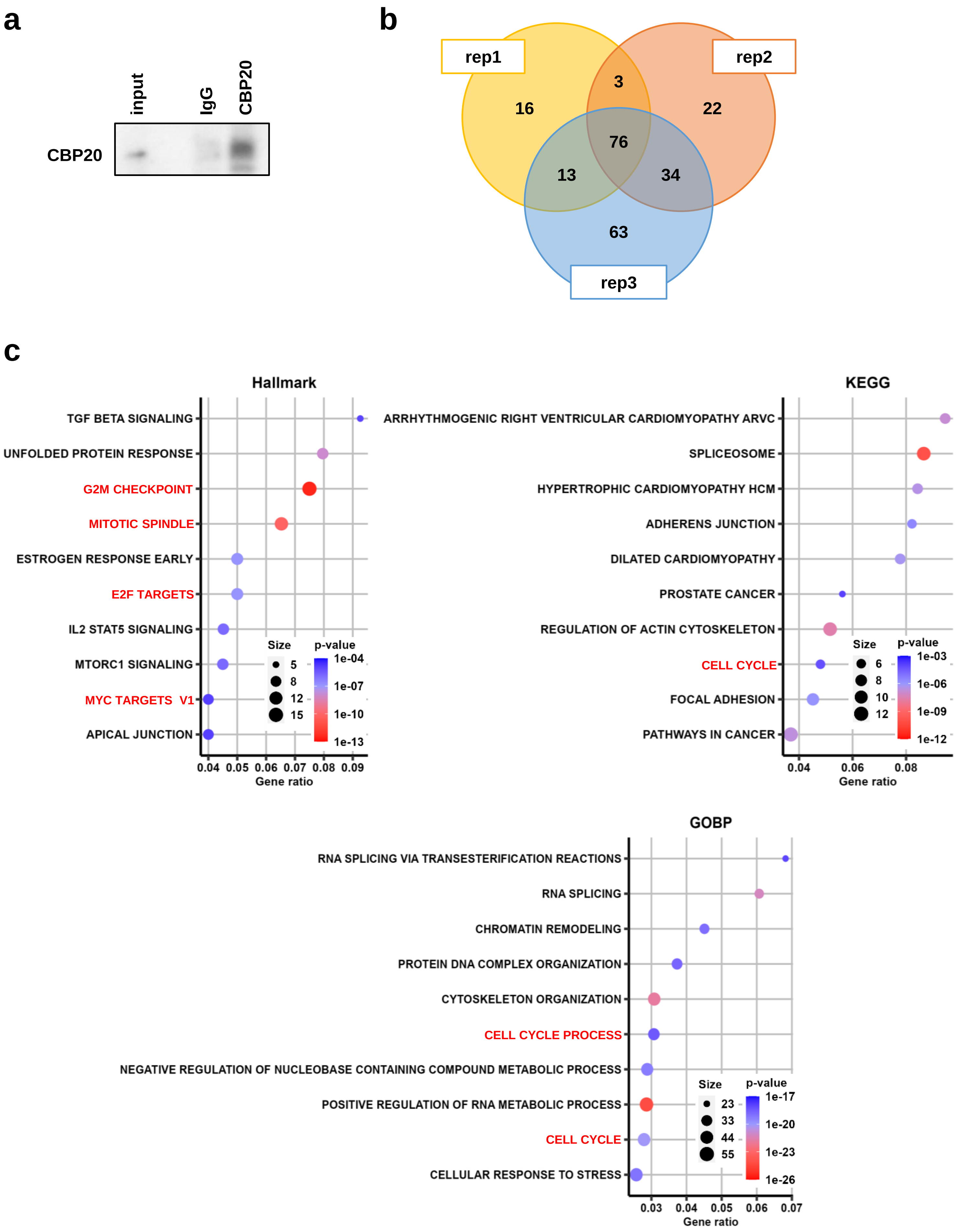

Supplementary Fig. 8 CBP20-bound mRNA genes were enriched to cell cycle related pathways.

a Western blot of CBP20 protein in A549 cells after RNA immunoprecipitation (RIP)-seq to validate immunoprecipitation efficiency. IgG was used as a control antibody. RIP-seq was performed in triplicate, and the figure represents a representative replicate.

b Venn diagram for annotated genes in each replicate after peak calling.

c Hallmark, KEGG, and GOBP pathway analysis for a total of 227 genes. The union of each replicate was used as input data for GSEA to investigate human gene sets, and cell cycle-related pathways are highlighted in red.

Supplementary Fig. 9

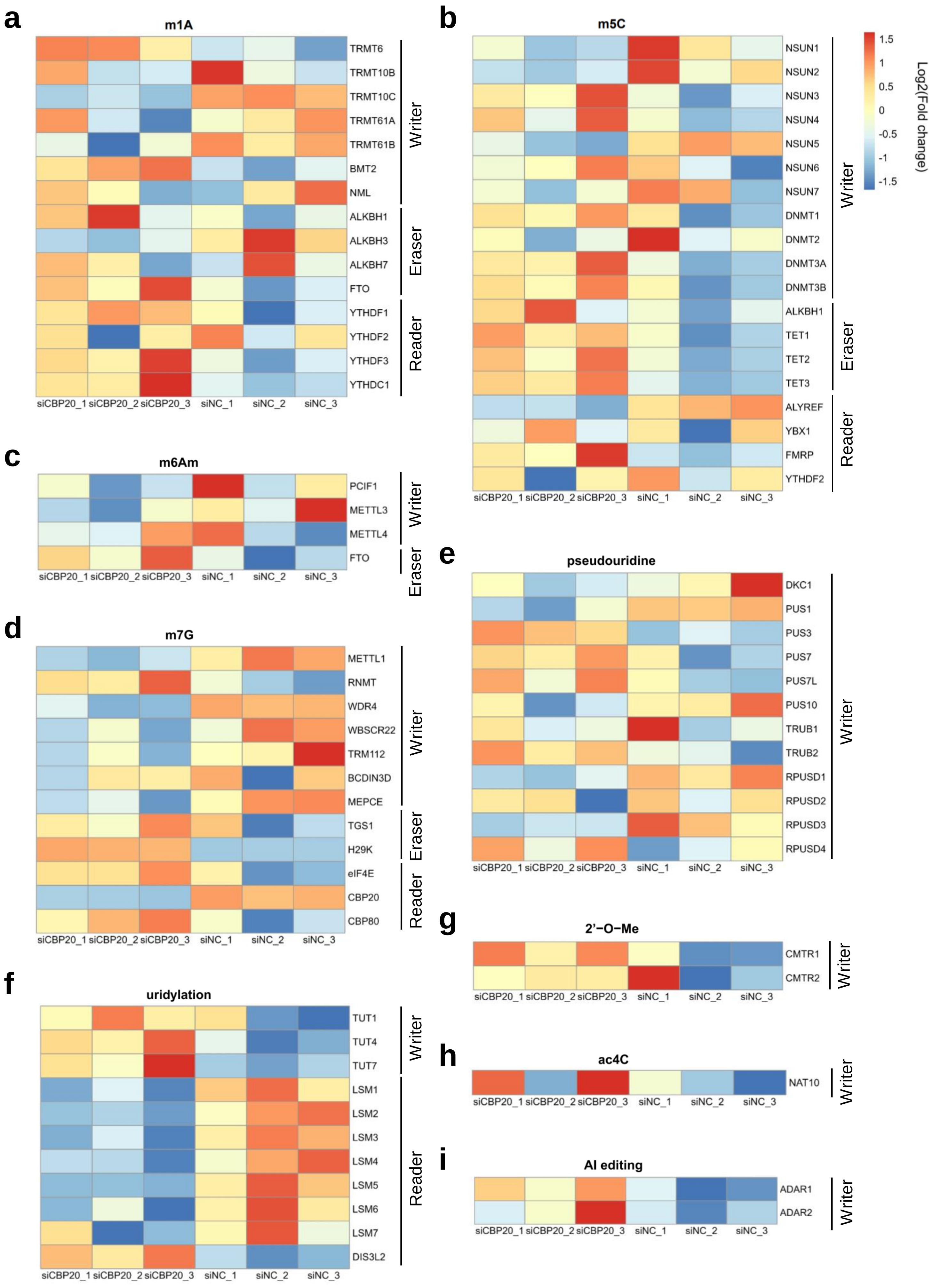

Supplementary Fig. 9 Effect of CBP20 depletion on the expression of RNA modification writers, erasers, and readers in RNA sequencing.

a-i Heatmap for mRNA expression of RNA modification-related genes from RNA sequencing results after CBP20 knockdown in A549 cells. Each column represents cells with negative control siRNA (siNC) or siRNA for CBP20 (siCBP20) and each row corresponds to RNA modification-related genes. The heatmap displays the Z-scores of RNA modification-related genes based on TPM values. The siCBP20 group is shown in the left three columns, while the siNC group is represented in the right three columns. Box colors indicate the corresponding Z-score. The RNA modification-related genes are categorized as follows: m1A, N^{1}-methyladenosine (a); m5C, 5-methylcytosine (b); m6Am, N^{6},2'-O-dimethyladenosine (c); m7G, N^{7-}methylguanosine (d); pseudouridine (e); uridylation (f); 2'-O-Me, 2'-O-methylation (g); ac4C, N^{4}-acetylcytidine (h); and AI editing, adenosine-to-inosine editing (i).

Supplementary Fig. 10

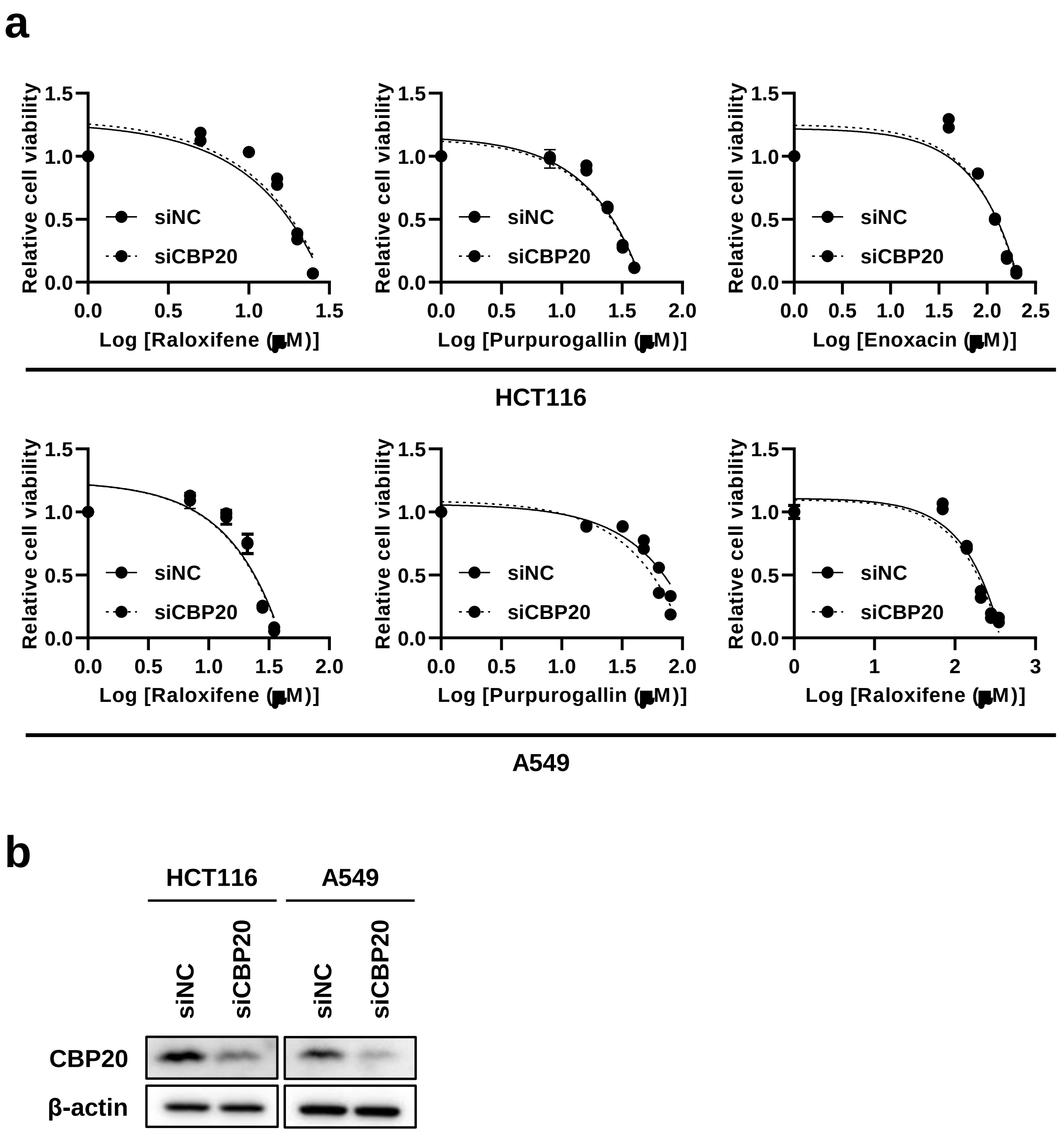

Supplementary Fig. 10 Effects of CBP20 depletion-mimicking chemicals after CBP20 knockdown in HCT116 and A549.

a Effect of raloxifene, purpurogallin, or enoxacin on HCT116 and A549 cells after CBP20 knockdown. Transfected cells were reseeded after transfection with siRNAs for 48 hours. Cytotoxicity for each drug measured by WST after exposing the cells to the drug for 72 hours. All graphs are represented as mean ± SD of experiments performed in triplicate.

b Western blot of CBP20 protein in HCT116 and A549 cells to validate CBP20 knockdown by siRNAs. Samples were obtained after transfection for 48 hours. β-actin was used as a loading control.

Supplementary Fig. 11

a

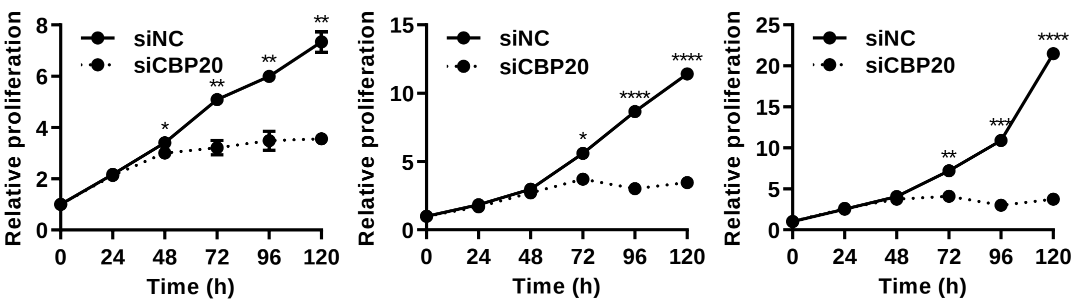

b

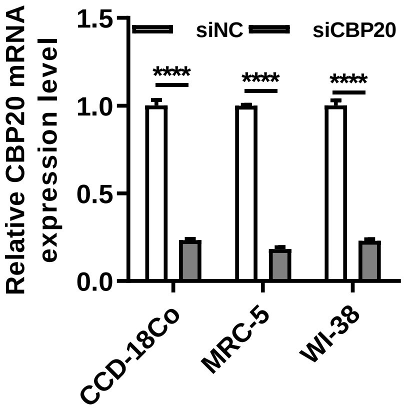

c

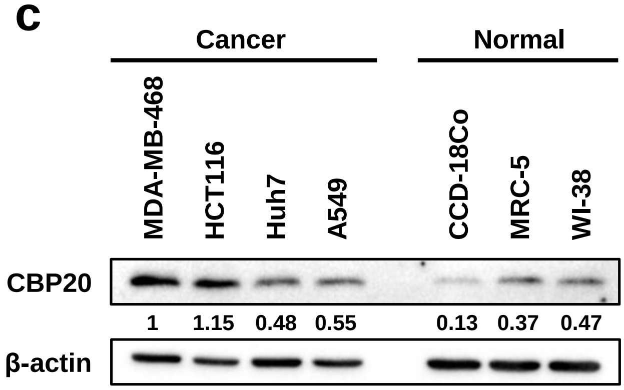

d

IC_{50}(μM)	Cancer cells		Normal cells		
	HCT116	A549	CCD-18Co	MRC-5	WI-38
Raloxifene	14.6958	21.9214	17.2662	17.4605	14.9689
Purpurogallin	28.5515	68.6379	786.085	69.8181	147.118
Enoxacin	89.4568	159.283	262.347	213.397	188.977

e

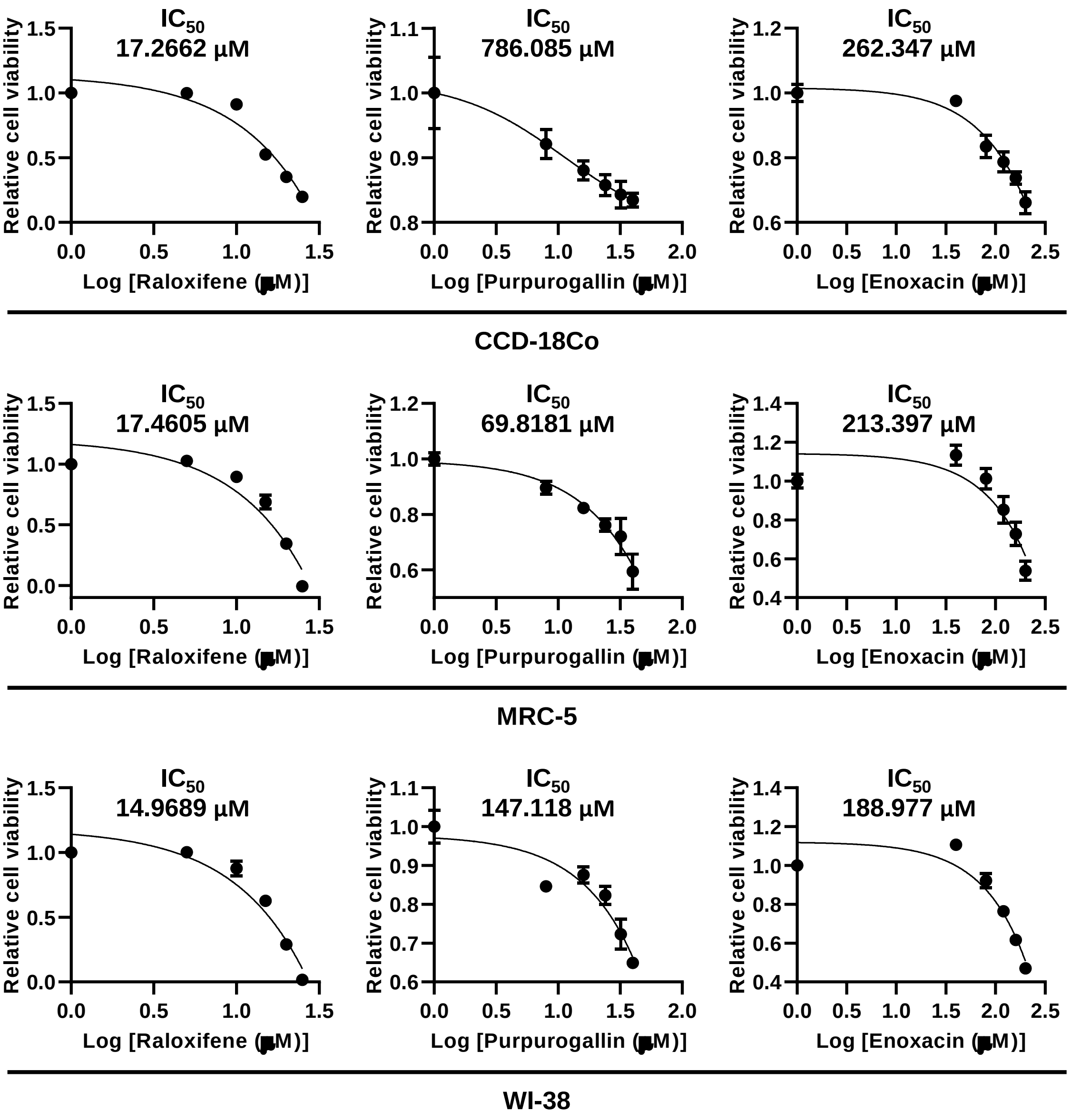

Supplementary Fig. 11 CBP20 depletion impairs normal cell proliferation while demonstrating enhanced drug sensitivity in cancer cells with elevated CBP20 expression.

a Proliferation of CCD-18Co, MRC-5, and WI-38 cells after 96-well transfection with negative control siRNA (siNC) or siRNA for CBP20 (siCBP20) for indicated time points. Cell proliferation was determined using WST assays after transfection of siRNAs.

b Validation of CBP20 knockdown by siRNAs in CCD-18Co, MRC-5, and WI-38 cells. Quantitative real-time PCR (qRT-PCR) was performed for CBP20 mRNA in three cell lines harvested from cell proliferation assay at 48 hours.

c Western blot of CBP20 protein in four cancer cell lines and three normal cell lines. β-actin was used as a loading control and band intensities were calculated using ImageJ.

d Summary table of IC_{50} values for three drugs across seven cell lines.

e Effect of raloxifene, purpurogallin, or enoxacin on normal cell viability. Cytotoxicity for each drug measured by WST after exposing the cells to the drug for 72 hours in CCD-18Co, MRC-5, and WI-38 cells. IC_{50} values were calculated using CompuSyn.

All graphs are represented as mean ± SD of experiments performed in triplicate. All p values were calculated by t-test ($p \geq 0.05$: ns, $p < 0.05$: *, $p < 0.01$: **, $p < 0.001$: ***, $p < 0.0001$: ****).

Supplementary Fig. 12

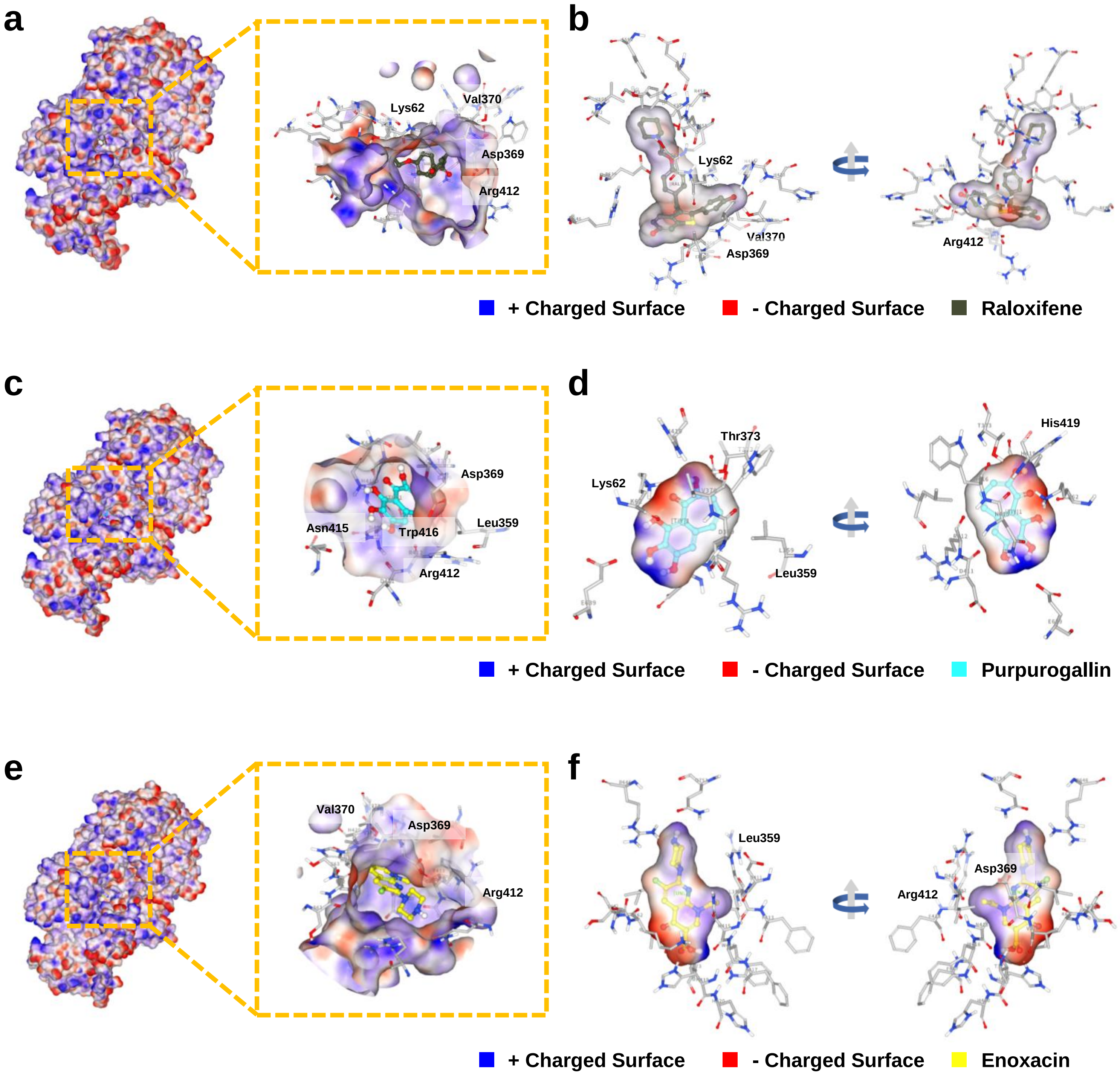

g

Rank	Drug	Predicted Binding Energy (kcal/mol)	Predicted Binding Affinity (nM)
1	Raloxifene	-5.41	86.7
2	Purpurogallin	-3.95	274.7
3	Enoxacin	-4.73	276.0

Supplementary Fig. 12 In-silico docking analysis of CBP20/CBP80 complex with CBP20 depletion-mimicking chemicals.

a Magnified view of the active site of 1H6K (complex formation with CBP20 and CBP80 protein) and raloxifene bound to the active site.

b 3D visualization of interactions between 1H6K and raloxifene.

c Magnified view of the active site of 1H6K and purpurogallin bound to the active site.

d 3D visualization of interactions between 1H6K and purpurogallin.

e Magnified view of the active site of 1H6K and enoxacin bound to the active site.

f 3D visualization of interactions between 1H6K and enoxacin.

g Summary table of predicted binding energy and predicted binding affinity of raloxifene, purpurogallin, and enoxacin to CBP20/CBP80 complex.

All AI-predicted ESP, and key residues were generated using the InteractionViewer module of the Pharmaco-Net platform.
